# Transcriptome Analyses of Near Isogenic Lines Reveal Putative Drought Tolerance Controlling Genes in Wheat

**DOI:** 10.3389/fpls.2022.857829

**Published:** 2022-03-29

**Authors:** Sina Nouraei, Md Sultan Mia, Hui Liu, Neil C. Turner, Guijun Yan

**Affiliations:** ^1^UWA School of Agriculture and Environment, The University of Western Australia, Perth, WA, Australia; ^2^The UWA Institute of Agriculture, The University of Western Australia, Perth, WA, Australia; ^3^Department of Primary Industries and Regional Development, Northam, WA, Australia

**Keywords:** drought stress, QTL, RNA-seq, NILs, SNP, DEGs, qRT-PCR, chromosome 4B

## Abstract

Drought stress, especially at the grain-filling stage, is a major constraint for wheat production. Drought tolerance is a complex trait controlled by a large array of genes and pathways. This study conducted gene expression profiling on two pairs of near-isogenic lines (NILs) for an important *qDSI.4B.1* QTL conferring drought tolerance on the short arm of chromosome 4B in wheat. Analysis showed 1,614 genome-wide differentially expressed genes (DEGs) between the tolerant and susceptible isolines in both NIL pairs. Six common DEGs were found between NIL1 and NIL2 at both 7 and 14 days after stress induction, with two of them having single nucleotide polymorphism (SNP) variants. These six genes that were confirmed by quantitative real-time PCR (qRT-PCR) expression analysis are considered candidate genes for drought tolerance mediated by *qDSI.4B.1* QTL with their main contributions to gene regulation, cell elongation, protein quality control, secondary metabolism, and hormone signaling. These six candidate genes and the highest number of DEGs and variants (SNPs/indels) were located between 49 and 137 Mbp of 4BS, making this interval the most probable location for the *qDSI.4B.1* locus. Additionally, 765 and 84 DEGs were detected as responsive genes to drought stress in tolerant and susceptible isolines, respectively. According to gene ontology (GO), protein phosphorylation, oxidation reduction, and regulation of transcription were top biological processes involved in the drought response and tolerance. These results provide insights into stress responses regulated by the 4BS locus and have identified candidate genes and genetic markers that can be used for fine mapping of the *qDSI.4B.1* locus and, ultimately, in wheat breeding programs for drought tolerance.

## Graphical Abstract

The workflow of this study. **(1)** Near isogenic lines for qDSI.4B.1 QTL responsible for drought tolerance were grown in control and drought stress conditions, **(2)** grain samples were collected at 7 and 14 days after stress initiation at anthesis, **(3)** RNA was extracted, **(4)** sequenced and **(5)** data were analysed and organized with focus on 4BS, **(6)** six candidate genes were found for drought tolerance in qDSI.4B.1 interval and sequencing results were confirmed by qRT-PCR, **(7)** the protein products and **(8)** molecular function of the candidate genes were future studied.

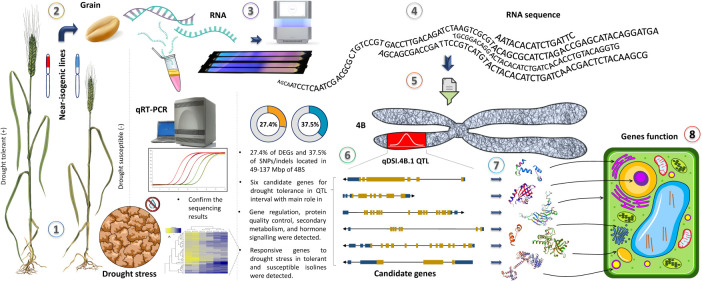

## Highlights

- Two NILs with contrasting response to drought stress were confirmed and analyzed by transcriptomics.
- Six candidate genes responsible for drought tolerance targeting the major locus were found and validated by qRT-PCR expression analysis.
- The *qDSI.4B.1* locus was deliminated to an interval of 49–137 Mbp on the short arm of chromosome 4B.

## Introduction

Wheat (*Triticum aestivum* L., 2*n* = 6*x* = 42, AABBDD) plays a key role in human nutrition by providing 20% of our dietary calories and proteins (Poole et al., 2021). With annual world production of 760.8 million tons in 2019, wheat is the most widely grown crop globally (Food and Agriculture Organization [FAO], 2021). The human population is predicted to surpass nine billion by 2050, which will increase the demand for wheat by 60%; to feed this population, the average wheat yield increases should be accelerated from current 1% per year to a minimum of 1.6% (Food and Agriculture Organization [FAO], 2017; Khadka et al., 2020).

Drought is among the most severe constraint of all biotic and abiotic stresses, thereby limiting crop productivity of dryland farming and threatening world food security (Zhang et al., 2018). Wheat is sensitive to water shortage, especially at the flowering and grain development stages, which can severely reduce the yield and grain quality (Kulkarni et al., 2017). In response to drought stress, wheat demonstrates various morphological, physiological, biochemical, and molecular responses through altered gene expression, and understanding of these mechanisms is necessary to improve adaptation of wheat varieties to drought-prone environments (Nezhadahmadi et al., 2013). However, the polygenic nature of the stress tolerance, controlled by many genes that have only small effects and high genotype by environment interactions (G × E), makes the understanding of the drought tolerance at the physiological and molecular levels very complex (Sallam et al., 2019). Additionally, hexaploid wheat has a large genome (17 GB of sequences and ∼128,000 genes), five times greater than the human genome, with 80–85% consisting of repetitive sequences that add to this complexity (Montenegro et al., 2017).

Several quantitative trait loci (QTLs) have been reported for grain yield and yield components under water stress on chromosomes 2BS, 2DS, 4AS, 4AL, 4BS, 6AS, 6BL, 7AL, and 7BL of wheat (Mathews et al., 2008; Alexander et al., 2012; Zaynali Nezhad et al., 2012; Mwadzingeni et al., 2016). To investigate the functional alleles of genes underlying QTLs, the mapping approach should be complemented by functional characterization (Habib et al., 2018). RNA sequencing (RNA-seq) is a highly sensitive transcriptomic approach that can be efficient in finding differentially expressed genes (DEGs). Moreover, this technique can be used to detect single nucleotide polymorphism (SNP) and insertion-deletion (indels) variants in transcribed genes that co-locate with a target locus (Ma et al., 2014). The variant calling not only provides information about the candidate genes but also can be used for developing molecular markers (Zhao et al., 2019). This information can lead to finding the molecular mechanisms and biosynthetic pathways involved in drought response and tolerance, especially in hexaploid wheat where multiple homeologous alleles exist for most genes and transcripts (Ma et al., 2014).

Typically, functional studies compare two genetically distinct lines (i.e., from different parents) with contrasting drought tolerance (Mia et al., 2020; Pereira et al., 2020; Chu et al., 2021). However, the various genetic backgrounds of tolerant and susceptible germplasm at many genomic locations make the accurate identification of candidate genes underpinning the QTL very difficult (Habib et al., 2018). The possible solution is to use lines with common genetic backgrounds but contrasting levels of drought tolerance, which can be achieved by developing near-isogenic lines (NILs) (Moumeni et al., 2011). NILs with identical genetic backgrounds, except for one or a few genetic locus/loci of interest, minimize the interference of the genetic background and enhance the sensitivity and accuracy of transcriptional analysis (Yan et al., 2017; Mia et al., 2019). The combined use of NILs with RNA-seq analysis was successfully used to identify resistance conferring genes, underpinning the QTL for important quantitative traits such as *Fusarium* crown rot, pre-harvest sprouting, and heat and drought stress in wheat (Ma et al., 2014; Schmidt et al., 2020; Wang et al., 2021).

A consistent major genomic region *qDSI.4B.1*, responsible for up to 22% of phenotypic variation under drought stress, has been reported on the short arm of chromosome 4B (4BS) around 27 Mbp away from the *Rht1* gene responsible for reduced height (Kadam et al., 2012). Additionally, Liu et al. (2020) have shown several consistent meta-QTLs for yield and yield components on 4BS that makes this location even more interesting for further scrutiny. In this study, we conducted a transcriptomic study through RNA-seq on two NIL pairs that were developed for a major effect genomic region, *qDSI.4B.1*, associated with drought tolerance on 4BS. The goals of this study were to (I) find genes responsive to drought stress in tolerant and susceptible isolines; (II) detect possible candidate genes responsible for drought tolerance within the *qDSI.4B.1* locus on 4BS; (III) determine the expression pattern, molecular function, and pathway for the detected genes that are (a) responsive to drought stress and (b) putatively responsible for drought tolerance in 4BS; and (IV) delineate the most probable interval for the *qDSI.4B.1* locus according to the DEGs and genes containing variants (SNPs and/or indels) on 4BS.

## Materials and Methods

### Development of Near Isogenic Lines

In a previous 2-year QTL mapping study, Kadam et al. (2012) mapped an important and consistent genomic region for drought tolerance (*qDSI.4B.1*) on the short arm of chromosome 4B in a mapping population of recombinant inbred lines (RILs). This QTL contribute to drought susceptibility index (DSI), grain yield (GY), shoot biomass (SB), root biomass (RB), harvest index (HI), and plant height (PH) under drought stress with a positive allele from wheat cultivar C306 (RGN/CSK3//2*C591/3/C217/N14//C281) (Kadam et al., 2012). Four confirmed NIL pairs of qDSI.4B.1-2, qDSI.4B.1-3, qDSI.4B.1-6, and qDSI.4B.1-8 were developed from a cross between C306 and Dharwar Dry following the heterogeneous inbred family (HIF) analysis, coupled with an immature embryo culture-based fast generation technique. Marker-assisted selection by the SSR marker of gwm368 linked to the *qDSI.4B.1* was carried out to identify heterozygous individuals for the targeted QTL in each generation, and NIL pairs were selected at F8 (Mia et al., 2019).

### Plant Material, Growth Condition, Stress Induction, and Sampling

To avoid the effect of the *Rht-B1* gene, which is closely located to the target locus, two NIL pairs with no height differences were selected for this study. Two NIL pairs of qDSI.4B.1-3 and qDSI.4B.1-8 (hereafter termed NIL1 and NIL2, respectively) were grown in a complete randomized block design with three biological replications from July to October of 2020 in a temperature-controlled and naturally lit glasshouse facility at The University of Western Australia (31°59′S, 115°49′E, and 31.5 m above the sea level) in Perth, Western Australia. Seeds were sown at 2.5-cm depth in cylindrical columns (a 9-cm diameter × 45-cm height) containing a 1.3-kg air-dried potting mix (5:2:3 fine composted pine bark: cocopeat: brown river sand, pH∼6.0) and 2.5-cm gravel at the base. The pot capacity (PC) of 100% was achieved by wetting up the soil and letting the pots freely drain for 48 h (Turner, 2019). The soil water content of the potting mix at PC was 38% using the following formula: $\%soilwatercontent=\frac{FW-DW}{DW}\times 100$, where FW and DW are the fresh and dry weight of the soil samples, respectively (Shemi et al., 2021). Pots were kept between 80 and 100% of PC by weighing from sowing (GS00) to anthesis (GS61), according to Zadoks growth scale for cereals (Zadoks et al., 1974). Two water treatments, well watered (WW) and drought stress (DS), were applied at the onset of anthesis (GS61). In the WW treatment, soil water content was kept between 80 and 100% of PC by watering at least every 2 days; while the DS treatment was applied by stopping irrigation for 14 days. Grains were collected at 7 days (7 d) and 14 days (14 d) of treatment application from DS and 7 days of treatment from WW treatment (named control), and then frozen immediately in liquid nitrogen and stored at −80°C for RNA extraction. The soil water content in the DS treatment was, on average, 42% and 24% of PC at 7 and 14 days post-anthesis, while it was 89% of PC in the WW treatment at sampling time. The soil water content was similar for tolerant and susceptible isolines at both 7 and 14 days after stress initiation. All pots were re-irrigated after sampling and kept at 80-100% of PC until physiological maturity.

### Morphological and Physiological Measurements at Time of Sampling

Chlorophyll fluorescence (Fv/Fm) was measured using a pocket PEA chlorophyll fluorimeter (Hansatech Instruments Ltd; Norfolk; United Kingdom). The flag leaf on the main stem was dark adapted for 0.5 h prior to measurement using the leaf clips. A SPAD-502 Plus chlorophyll meter (Konica Minolta, Osaka, Japan) was used to read the SPAD values of the flag leaf of the main stem, and raw reads were used as a comparison for chlorophyll content in resistant and susceptible isolines. Plant temperature was measured on the main stem flag leaf using an Impac IGA 15 plus with a laser-targeting light (Advanced Energy, Colorado, United States). At the same time, the ambient temperature was recorded by a digital thermometer, and differences between plant and ambient temperature were reported. The relative water content (RWC) was measured around midday of the sampling dates, following the method described by Turner (1981). Other traits, including days from sowing to maturity, plant height, peduncle length on the main stem, flag leaf sheath length, fertile tiller number, spike length on the main stem, spikelet number on the main spike, fertile spikelet number on the main spike, awn length on the main spike, flag leaf length on the main stem, dry weight of aerial parts, 1,000-kernel weight, and grain yield (per plant), were evaluated at physiological maturity and compared by *t*-test between the two isolines in both NIL pairs.

### RNA Extraction, Library Construction, Sequencing, and Quality Control

To isolate pure high-quality total RNA from 36 seed samples (4 genotypes × 3 treatments and time point × 3 biological replications), a TRIzol plus RNA purification kit (Invitrogen, MA, United States), in combination with ISOLATE II RNA plant kit (Meridian Bioscience, United States), with an on-column DNase I treatment, was used as described by Furtado (2014). The purity (Absorbance at 260/280 and 260/230 nm) and concentration of extracted RNA was assessed by NanoDrop 2000 (Thermo Fisher Scientific Inc., CA, United States) and Qubit 4 Fluorometer (Invitrogen, MA, United States). The integrity was checked by LabChip GX (PerkinElmer, MA, United States), measuring RNA integrity number (RIN). The RNA samples were then sent to the Australian Genome Research Facility (AGRF) (Parkville, Victoria, Australia) for sequencing. Initially, mRNAs were isolated from total RNA by oligo (dT) beads and used for cDNA synthesis, and then 150-bp paired-end sequencing libraries were produced and sequenced through Illumina NovaSeq 6000 (Illumina, San Diego, United States). The primary sequence data were generated in FASTQ format using the Illumina bcl2fastq v2.20 pipeline. Raw reads were trimmed using Trim Galore v0.4.4 with a minimum Phred quality value of 30 and minimum final read length of 70 bp, and also screened for the presence of any Illumina adapter, overrepresented sequences, and cross-species contamination (Cox et al., 2010). The ‘‘clean data’’ for the 36 libraries are publicly available at the Sequence Read Archive (SRA) of the National Center for the Biotechnology Information (NCBI) website with the accession number PRJNA760243^1^.

### Sequence Mapping and Differentially Expressed Gene Identification

The cleaned sequence reads were then aligned against the bread wheat reference genome, International Wheat Genome Sequence Consortium (IWGSC) RefSeq v1.0^2^, using STAR aligner v2.5.3a (Dobin et al., 2013; Rudi et al., 2018). The transcripts were assembled with the StringTie tool v2.1.4 using the reads alignment and reference annotation-based assembly option (RABT) to generate assembly for known and potentially novel transcripts (Kovaka et al., 2019). The mapped reads were annotated to features in the *T. aestivum* annotation file, and the gene expression level (gene counts) was calculated by the feature Counts v1.5.3 utility of the subread package (Liao et al., 2019). The edgeR v3.32.1 was used to detect and quantify DEGs according to the expression level of high-confidence genes in R v4.0.5 (R Core Team, 2020). The default TMM normalization method of edgeR was used to normalize the counts between samples, and a generalized linear model was then used to quantify the differential expression between the groups. Fragments per kilobase of exon per million mapped reads (FPKM) was calculated for each transcript to represent the normalized expression value. The fold change in gene expression was calculated according to the equation: Fold change = log_2_ (FPKM_A_/FPKM_B_). DEGs were determined with the threshold of log2fold change of ≥1 or ≤−1 and false discovery rate (FDR) of ≤0.05.

Pairwise comparisons were conducted in two ways: firstly, between different treatments for the same isoline to find genes responsive to drought stress: T^7d^_v_T^C^; T^14d^_v_T^7d^; S^7d^_v_S^C^; S^14d^_v_S^7d^; and secondly, between isolines under drought stress condition to find genes putatively responsible for drought tolerance: T^7d^_v_S^7d^; T^14d^_v_S^14d^ (Supplementary Figure S1). Symbols are “C” for control; “T” for tolerant; “S” for susceptible; “7d” and “14d” for 7 and 14 days after stress imposition, respectively; and “A_v_B” for comparing object “A” with “B,” i.e., if a gene expression in “A” was higher or lower than that in “B,” it was considered upregulated or downregulated, respectively.

### Functional Annotations and Pathway Enrichment

Gene ontology (GO) and plant reactome pathway analysis were performed for the DEGs identified from all comparisons. For the GO analysis, the bioMart v0.7 was used to identify GO terms corresponding to the DEGs. The two data frames were used as input in edgeR v3.32.1 to find the most highly enriched GO terms with the threshold of *p*-value < 0.05, and the top 20 for each of the three ontologies in the GO results were plotted (Gene Ontology Consortium, 2021). The reactome analysis was carried out through pathway browser v3.5 by following the user guide in the Plant Reactome v20 database (Naithani et al., 2020). The overall top 30 pathways with a false discovery rate (FDR)<0.05 were plotted in the plant reactome results. The plots were created using the ggplot2 v3.3.3 package in R v4.0.5 (R Core Team, 2020).

### Identification of Single Nucleotide Polymorphism and Indel Variants

To identify variants between T and S isolines, all nine sequence files for each isoline (three biological replication by three treatments) were combined after removing low-quality sequences. The Samtools v1.9 was used to create a pileup of reads against the reference genome from each BAM file (Binary Alignment Map of the reads) that was already generated by STARaligner v2.5.3a. Then, the bcftools v1.9 compares the pileup data against a reference genome to identify variants (SNPs or indels) (Danecek et al., 2021). The bcftools isec was used to filter only those variants that were present in all samples of each isoline, and J browser v1.16.3 was used to annotate the variants on the 4BS chromosome against the International Wheat Genome Sequence Consortium RefSeq v1.0 see text footenote 2 (Buels et al., 2016).

### Validation of Differentially Expressed Genes Using Quantitative Real Time PCR

Six candidate genes for drought tolerance were selected to be validated with quantitative real-time PCR (qRT-PCR). Gene-specific (an exon-exon junction) primers were designed by PrimerQuest Tool (Integrated DNA Technologies, IA, United States), and the actin protein gene was used as an internal housekeeping reference for normalization between samples. RNA was extracted with the method described previously, and three biological replications in two separate wells (technical replication) were applied. The cDNA was synthesized by SensiFAS cDNA Synthesis Kit (Meridian Bioscience, United States), and qRT-PCR was performed on ABI 7500 Fast Real Time PCR (Applied Biosystems, CA, United States) using the SensiFAST SYBR Lo-ROX Kit (Meridian Bioscience, United States), following methods described by Wang et al. (2021). The relative fold changes were calculated using the comparative CT method (2^–ΔΔCT^). The average value of the two technical replications was considered for each biological replication.

## Results

### Effect of Drought Stress on Morphological and Physiological Differences Between Near-Isogenic Lines Isolines

**FIGURE 1:**
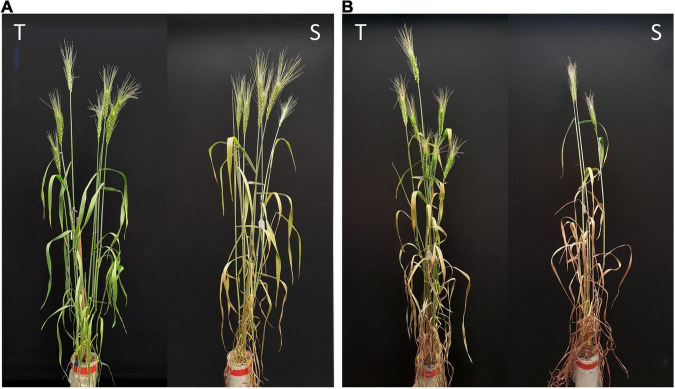
Representative wheat plants of the tolerant (T) and susceptible (S) near isogenic lines of NIL1 at panels **(A)** 7 days and **(B)** 14 days after drought stress initiation at anthesis.

Drought stress was effective in reducing the leaf RWC by more than 40% after 7 days and more than 70% after 14 days without water. The comparison between tolerant (+NIL) and susceptible (−NIL) isolines showed significant differences in chlorophyll content, chlorophyll fluorescence, RWC, dry weight of aerial parts, 1,000-kernel weight, and grain yield under stress conditions in both NIL1 and NIL2 (Table 1). In control condition, the grain yield was 28.8 g in both isolines of NIL1, which was not significantly different between T and S isolines. However, after 7 days of drought stress, grain yield in T and S isolines was significantly different at 21.6 and 15.4 g per plant, respectively. After 14 days of drought stress, grain yield was also significantly different at 10.6 and 6.7 g per plant in T and S isolines, respectively. The same trend was also witnessed between T and S isolines in NIL2 and for other characteristics (Table 1). The more comprehensive data of morphological and physiological measurements are provided as Supplementary Data (Supplementary Table S1). As expected, the isolines carrying the tolerant allele from the donor parent C306 showed higher yields and yield-related traits than their counterparts, which confirmed they are true NIL pairs (Figures 1A,B).

**TABLE 1:** Comparison of yield and yield-related morphological and physiological characteristics (mean ± standard deviation) of near-isogenic lines under stress and non-stress conditions.

Isoline		Treat	Chlorophyll content (SPAD reads)	*p*	Chlorophyll fluorescence (Fv/Fm)	*p*	Relative water content (%)	*p*	Dry weight of aerial parts (g/plant)	*p*	1000-kernel weight (g)	*p*	Grain yield (g/plant)	*p*
NIL1	T	C	51.8 ± 1.02	**	0.76 ± 0.006	ns	87.6 ± 5.64	ns	112.0 ± 7.41	ns	57.5 ± 2.54	ns	30.3 ± 1.52	ns
	S	C	45.2 ± 0.98		0.78 ± 0.005		93.7 ± 1.20		120.0 ± 1.55		59.8 ± 0.92		27.4 ± 0.71	
NIL1	T	7d	44.7 ± 0.68	*	0.62 ± 0.003	ns	48.9 ±1.47	*	57.5 ± 0.95	*	34.8 ± 1.52	*	21.6 ± 1.47	*
	S	7d	40.8 ± 0.42		0.57 ± 0.030		40.6 ± 2.16		53.8 ± 0.78		28.4 ± 1.53		15.4 ± 1.47	
NIL1	T	14d	10.5 ± 1.07	*	0.38 ± 0.012	*	20.7 ± 1.46	ns	61.3 ± 1.42	*	19.7 ± 0.95	*	10.6 ± 1.21	*
	S	14d	5.3 ± 0.60		0.31 ± 0.014		19.1 ± 1.30		44.4 ± 3.01		14.3 ± 1.47		6.7 ± 0.42	
NIL2	T	C	54.3 ± 0.33	ns	0.77 ± 0.009	ns	87.8 ± 0.65	ns	89.2 ± 5.42	ns	58.3 ±1.62	ns	26.3 ± 1.24	ns
	S	C	55.4 ± 0.21		0.77 ± 0.015		86.5 ±1.08		90.9 ± 3.67		53.7 ± 1.16		23.9 ± 0.71	
NIL2	T	7d	54.0 ± 1.12	**	0.68 ± 0.023	*	46.0 ± 1.38	*	39.1 ± 0.96	*	36.3 ± 0.82	*	15.5 ± 0.43	*
	S	7d	45.2 ± 0.64		0.59 ± 0.023		40.4 ± 0.53		35.1 ± 0.69		32.1 ± 1.11		13.6 ± 0.29	
NIL2	T	14d	12.2 ± 0.69	*	0.44 ± 0.026	*	25.8 ± 0.63	*	25.5 ± 0.99	*	18.3 ± 0.38	*	6.9 ± 0.18	**
	S	14d	8.6 ± 0.70		0.27 ± 0.044		20.6 ± 1.22		20.7 ± 0.59		16.2 ± 0.50		4.9 ± 0.30	

*“NIL1” is qDSI.4B.1-3 pair, and “NIL2” is qDSI.4B.1-8 pair.*

*“T” and “S” represent tolerant and susceptible isolines, respectively.*

“C” stands for control, “7d” for 7 days after drought stress imposition at anthesis, “14d” for 14 days after drought stress imposition at anthesis, “Treat.” for Treatment, “p” for p-value. ns = non-significant, * = significant at p ≤ 0.05, ** = significant at p ≤ 0.01.

### Transcriptome Assembly and Mapping Quality

A total of 3.26 billion high-quality 150-bp paired-end reads equal to 985 Gb of data were generated from the 36 samples. The quality of reads looked excellent, with >92% of bases had a quality score of Q30. Approximately, 80% of the reads were mapped to the wheat genome, including 69% with a unique match (Supplementary Figure S2 and Supplementary Table S2).

According to the multidimensional scaling plot (MDS), the highest distance that corresponds to the leading log fold change of RNA-sequencing samples was between treatments, and lowest distance was between replications. The distance between 14 days and 7 days was higher than 7 days and control in both NIL pairs. The difference between NIL isolines was more than between replications and less than the treatments, which shows that the experiment was well controlled and worked well (Supplementary Figure S3).

### Genes Induced by Drought Stress

**FIGURE 2:**
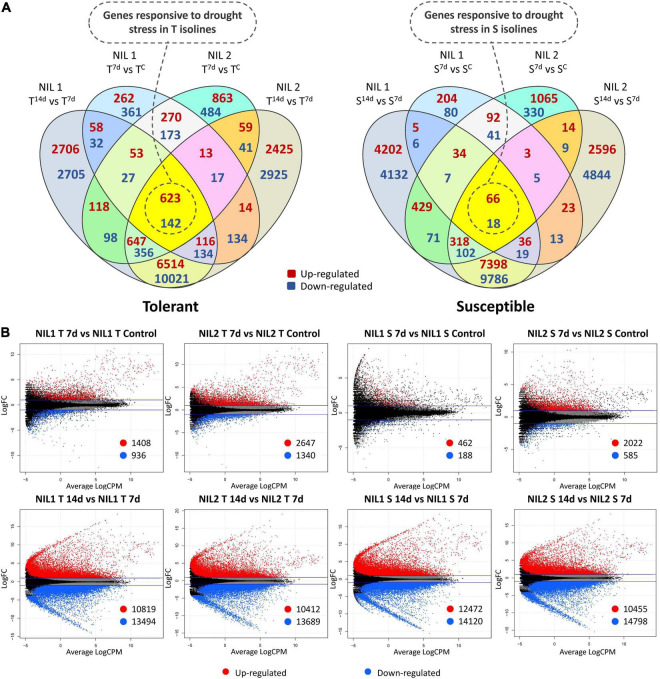
**(A)** Venn diagrams and **(B)** Smear plots for differentially expressed genes (DEGs) related to each of the isolines (T vs. T and S vs. S) at 7 days after drought stress initiation in comparison to control and 14 days in comparison to 7 days after stress. The *X*-axis is the average of log counts per million. The *Y*-axis is the log2 fold change. Symbols are “C” for control; “T” for tolerant isoline; “S” for susceptible isoline; “7d” and “14d” for 7 and 14 days after drought stress initiation at anthesis, respectively. The DEGs were detected with the threshold false discovery rate (FDR) of ≤0.05 and the absolute value of log2 fold change ≥1 or ≤−1.

We identified transcripts that were differentially expressed between control and stress conditions of the same isoline (T vs. T and S vs. S) as an indication of what molecular mechanisms may be associated in response to drought stress in each isoline (Figures 2A,B).

In the tolerant isolines, for NIL1, 2,344 DEGs (1,408 up and 936 downregulated) and, for NIL2, 3,987 DEGs (2,647 up and 1,340 downregulated) were detected in comparison between 7 days after stress and control conditions (T^7d^_v_T^C^). In the comparison of 14 days with 7 days after stress induction, 10,819 DEGs were up and 13,494 were downregulated (total of 24,313) in the NIL1 tolerant isoline. Likewise, 10,412 DEGs were up and 13,689 were downregulated (total of 24,101) in the tolerant isoline of NIL2 (T^14d^_v_T^7d^) (Figure 2B). The 765 DEGs, including 623 upregulated and 142 downregulated genes, were common between all four comparisons of tolerant isolines (T_v_T) of NIL1 and NIL2 in comparisons between 7 days after stress induction to control and 14 days to 7 days after stress induction (Figure 2A (left) and Supplementary Table S3). These 765 DEGs were considered as genes responsive to drought stress in the tolerant isolines.

In the susceptible isolines, 462 DEGs were up and 188 downregulated (total of 650) when comparing 7 days after stress and the controls in NIL1. However, this number in the NIL2 susceptible isoline was 2,022 up and 585 downregulated DEGs (total of 2,607) for the same comparison (S^7d^_v_S^C^) (Figure 2B). In the comparison of 14 days with 7 days after stress imposition, in the NIL1 susceptible isoline, 26,592 DEGs (12,472 up and 14,120 downregulated) and, in NIL2 susceptible isoline, 25,253 DEGs (10,455 up and 14,798 downregulated) were detected (S^14d^_v_S^7d^) (Figure 2B). From all four comparisons of susceptible isolines (S_v_S) of NIL1 and NIL2 at both time point comparisons of 7 days to control and 14 days to 7 days after stress induction, 84 DEGs, including 66 upregulated and 18 downregulated, were common (Figure 2A (right) and Supplementary Table S3). These 84 DEGs were considered as genes responsive to drought stress in the susceptible isolines.

### The Function of Genes Induced by Drought Stress

**FIGURE 3:**
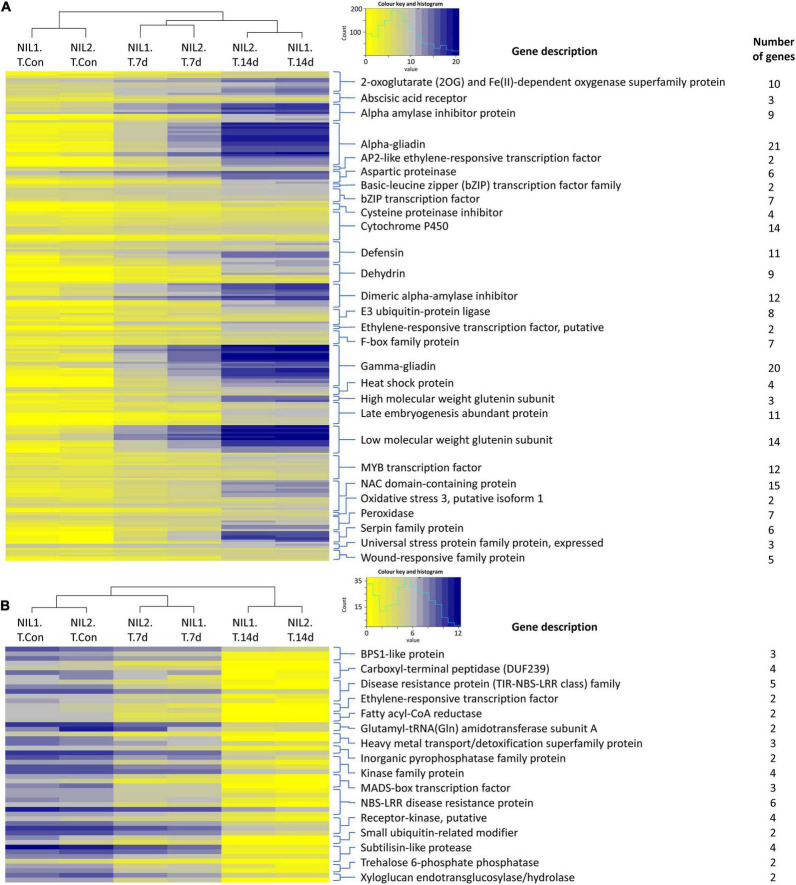
Heatmaps showing the expression of the important **(A)** upregulated and **(B)** downregulated genes responsive to drought stress in the tolerant isolines. Color keys represent the log2 of normalized expression values and a histogram of the counts. Each row represents a gene and each column a sample. Symbols are “Con” for control; “T” for tolerant isoline; “7d” and “14d” for 7 and 14 days after drought stress initiation at anthesis, respectively. The DEGs were determined with the threshold false discovery rate (FDR) of ≤0.05 and the absolute value of log2 fold change ≥1 or ≤−1.

The expression values for the important families of genes responsive to drought stress in the tolerant isolines are illustrated as heatmaps in Figure 3. The complete list of these genes, their functions, and expression are presented in Supplementary Table S3. In genes responsive to drought stress in tolerant isolines, the 623 upregulated genes contained 21 genes encoding alpha-gliadin, 20 encoding gamma-gliadin, 15 encoding NAC domain-containing protein, 14 encoding cytochrome P450, 14 encoding low molecular weight glutenin subunit, 12 encoding the MYB transcription factor, and 12 encoding the dimeric alpha-amylase inhibitor. These were the highest number of DEGs with the same encoding protein following the 11 encoding genes for each of the defensin and late embryogenesis abundant proteins, the 10 genes for 2-oxoglutarate (2OG) and Fe(II)-dependent oxygenase, nine for dehydrin, nine for alpha amylase inhibitor protein, and eight for E3 ubiquitin-protein ligase (Figure 3A). From the 142 downregulated genes, six genes encoded NBS-LRR disease resistance protein, five encoded the disease-resistance protein (TIR-NBS-LRR class) family, and four encoded the carboxyl-terminal peptidase, kinase family protein, receptor-kinase, and subtilisin-like protease (Figure 3B).

The expression values for the genes responsive to drought stress in susceptible isolines are illustrated as heatmaps in Supplementary Figure S4 and additional information in Supplementary Table 3. In genes responsive to drought stress in susceptible isolines, early nodulin 93 protein with 11 genes, and BTB/POZ and TAZ domain protein with three genes had the highest number of upregulated encoding DEGs. The two encoding genes were found for each of cytochrome P450, 70 kDa heat shock protein, calcium-binding EF-hand, phosphatidylethanolamine-binding protein, and pro-resilin (Supplementary Figure S4A). In the downregulated DEGs, trehalose 6-phosphate phosphatase, and MYB transcription factor, each had two encoding genes (Supplementary Figure S4B).

### Genes Putatively Responsible for Drought Tolerance

**FIGURE 4:**
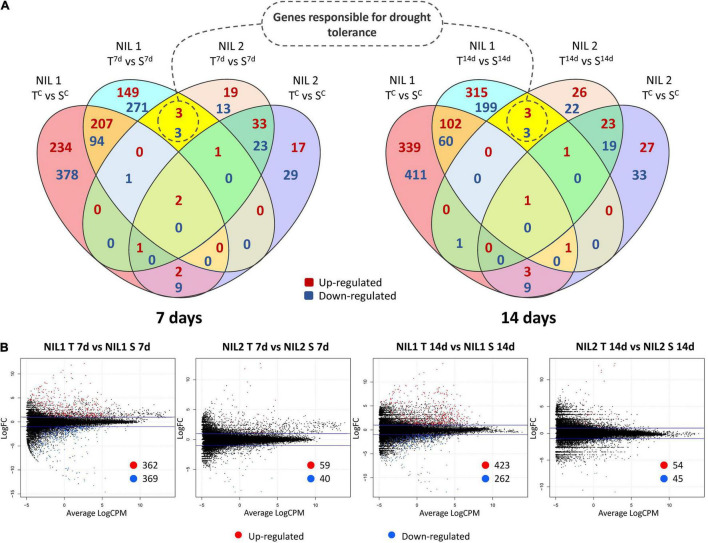
**(A)** Venn diagrams and **(B)** Smear plots for differentially expressed genes (DEGs) between the tolerant and susceptible isolines (T vs. S) at 7 and 14 days after drought stress initiation at anthesis. The *X*-axis is the average of log counts per million. The *Y*-axis is the log2 fold change. Symbols are “C” for control; “T” for tolerant isoline; “S” for susceptible isoline; “7d” and “14d” for 7 and 14 days after drought stress initiation at anthesis, respectively. The DEGs were determined with the threshold false discovery rate (FDR) of ≤0.05 and the absolute value of log2 fold change ≥1 or ≤−1.

**FIGURE 5:**
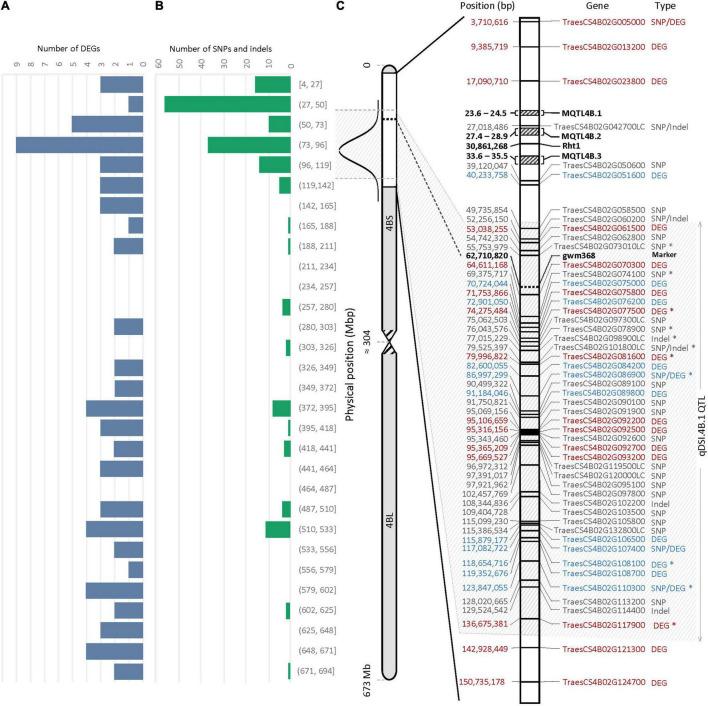
Physical distribution of panel **(A)** the differentially expressed genes (DEGs) **(B)** the single nucleotide polymorphisms (SNPs) and/or indels on chromosome 4B. **(C)** Physical distribution of DEGs and genes containing SNPs and/or indels within a 150-Mb interval of the 4BS. The most probable interval of the *qDSI.4B.1* QTL is shown between the arrows. Genes with SNPs and/or indels are shown in gray; The DEGs with higher expression in tolerant (T) and susceptible (S) isolines are shown in blue and red, respectively. Genes with an asterisk (*) were common between NIL1 and NIL2. *Rht1* is the reduced plant height gene; MQTL4B.1, MQTL4B.2, and MQTL4B.3 are major meta-QTLs for yield according to Liu et al. (2020).

An important part of this experiment was to identify transcripts associated with molecular mechanisms that might be responsible for drought tolerance meditated by *qDSI.4B.1* QTL. To this end, we identified transcripts that were differentially expressed between the tolerant and susceptible isolines (T vs. S) at 7 days and 14 days after stress induction with particular attention to the DEGs located on 4B chromosome. The comparative expression analysis detected the sum of 1,614 DEGs (898 upregulated and 716 downregulated) from all four comparisons between the T and S isolines (NIL1.T^7d^_v_S^7d^; NIL1.T^14d^_v_S^14d^; NIL2.T^7d^_v_S^7d^; NIL2.T^14d^_v_S^14d^) (Figure 4). The 73 unique DEGs mapped on the 4B chromosome showed high consistency in both NIL pairs. The largest portion (27.4%) of DEGs on 4B was located between 49 and 137 Mb of the short arm of the chromosome 4B (4BS) where the targeted QTL exists (Figure 5A).

At 7 days after stress induction, 731 DEGs (362 up and 369 downregulated) were detected between T and S isolines in NIL1. In NIL2, 99 DEGs (59 up and 40 downregulated) were identified between T and S isolines at 7 days after stress induction (Figures 4A(left),B). Of these DEGs, 33 in NIL1 and 10 in NIL2 were located on the 4B chromosome, with six in common between NIL1 and NIL2 (Table 2). At 14 days after stress induction, 423 DEGs were up and 262 were downregulated (total of 685) in comparison between T and S isolines in NIL1, while these numbers for NIL2 were 54 up and 45 downregulation DEGs with the total number of 99 (Figures 4A(right),B). Of these DEGs, 43 in NIL1 and 7 in NIL2 were located on 4B chromosomes, with six in common between NIL1 and NIL2 (Table 2). Overall, the six genes, including *TraesCS4B02G110300*, *TraesCS4B02G086900*, and *TraesCS4B02G108100* with higher expression in T, and *TraesCS4B02G077500*, *TraesCS4B02G081600*, and *TraesCS4B02G117900* with higher expression in S, were common DEGs between NIL1 and NIL2 interestingly at both 7 and 14 days after stress induction (Figure 4A(left and right)). These six genes were all located on 4BS and were not DEGs under control condition (NIL1.T^C^_v_S^C^ and NIL2.T^C^_v_S^C^); otherwise, their differences would be due to differences in a genetic background and not drought tolerance. Therefore, these six genes can be considered as important candidate genes putatively responsible for drought tolerance in the wheat *qDSI.4B.1* QTL (Figure 4A, Table 3, and Supplementary Table S4).

**TABLE 2:** The number of upregulated, downregulated, and total differentially expressed genes (DEGs) across the whole genome and on chromosome 4B from comparisons between tolerant (T) and susceptible (S) isolines in two NIL pairs (T vs. S).

		NIL pairs					
		NIL1		NIL2		Common	
Treatments	DEG	Genome	4B	Genome	4B	Genome	4B
7d	Up	362	24	59	5	6	3
	Down	369	9	40	5	4	3
	Total	731	33	99	10	10	**6**
14d	Up	423	23	54	3	5	3
	Down	262	20	45	4	3	3
	Total	685	43	99	7	8	**6**

*“7d” and “14d” represent 7 and 14 days after stress initiation, respectively.*

*“Up” stands for upregulated and “Down” for downregulated in tolerant relative to susceptible isolines, “Common” for common to both NIL1 and NIL2.*

**TABLE 3:** Candidate genes considered responsible for drought tolerance in the QTL *qDSI.4B.1* located on the short arm of chromosome 4B (4BS).

Higher exp. in	Gene ID	Physical position	Gene description	Pathway	Number of SNPs
S	TraesCS4B02G077500	74275484–74281641	Myosin-2 heavy chain-like protein	Unknown	0
S	TraesCS4B02G081600	79996822–80000363	B3 domain-containing protein	Unknown	0
T	TraesCS4B02G086900	86997299–87012097	Transducin/WD40 repeat-like superfamily protein, putative	Unknown	1 in intron
T	TraesCS4B02G108100	118654716–118680106	ATP-dependent protease La (LON) domain-containing protein	Unknown	0
T	TraesCS4B02G110300	123847055–123848467	Elongation factor Ts	Unknown	1 in exon
S	TraesCS4B02G117900	136675381–136680908	Signal recognition particle 54 kDa protein	Signal recognition particle subunit SRP54 (K03106)	0

*“exp.” stands for expression, “T” for tolerant, and “S” for susceptible.*

*“Higher expression in T” means upregulated in T compared to S, and “Higher expression in S” means downregulated in T compared to S.*

### Single Nucleotide Polymorphisms and Indels Variants Between Tolerant and Susceptible Isolines

According to variant calling analysis between the T and S isolines in both NIL pairs, 174 SNPs and 12 indels were detected on the 4B chromosome (110 variants came from NIL1 and 76 from NIL2). Similar to DEGs, the 4B chromosome showed high consistency in the number of variants in both NIL pairs, and the largest proportion (37.5%) of SNP and indel variants was located at 27 to 137 Mb of the short arm of the 4B chromosome (Figure 5B and Supplementary Table S5).

The 29 genes with variants inside were detected on 137 Mb of the 4BS chromosome, including *TraesCS4B02G005000*, *TraesCS4B02G086900*, *TraesCS4B02G107400*, and *TraesCS4B02G110300*, which were also found differentially expressed under stress in this study (Figure 5C). Two of these four DEGs, *TraesCS4B02G086900* and *TraesCS4B02G110300*, were common DEGs between both NIL pairs and could be the most important candidate genes in the *qDSI.4B.1* QTL responsible for drought tolerance in wheat (Figure 5C). *TraesCS4B02G005000* with higher expression in S had 13 SNPs in exon and one SNP in the 3′ untranslated region (UTR) encoding the NBS-LRR-like resistance protein. From 13 SNPs, ten were non-synonymous and three were synonymous SNPs. Non-synonymous SNPs change the amino acid sequence of protein, while synonymous SNPs do not affect the protein sequence. From ten non-synonymous SNPs, nine SNPs resulted in changes in the amino acid of the protein (a missense variant), and one SNP led to a stop codon (a nonsense variant) (Supplementary Table S5). *TraesCS4B02G086900* with higher expression in T had one SNP in intron and encodes the transducin/WD40 repeat-like superfamily protein. *TraesCS4B02G107400* and *TraesCS4B02G110300*, both with higher expression in T, each had one SNP in exon and encoded tapetum determinant 1 (TPD1) and elongation factor Ts protein, respectively. *TraesCS4B02G107400* had a non-synonymous SNP with different allelic variations, which corresponded to lysine in tolerant and glutamic acid in susceptible isoline. The synonymous SNP in *TraesCS4B02G110300* was in a residue overlap splice site and led to synthesis of arginine in both isolines (Supplementary Table S5).

### Functional Annotation of Differentially Expressed Genes

**FIGURE 6:**
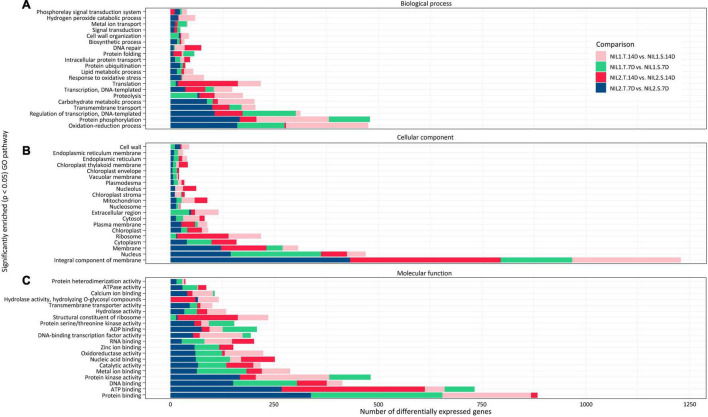
Gene ontology analysis (GO) of the differentially expressed genes (DEGs) identified when comparing tolerant and susceptible (T vs. S) isolines. Top significantly enriched pathways in panels **(A)** biological processes, **(B)** cellular components, and **(C)** molecular function are illustrated with *p-*value < 0.05. Symbols are “T” for tolerant isoline; “S” for susceptible isoline; “7D” and “14D” for 7 and 14 days after drought stress initiation at anthesis, respectively.

The GO analysis categorized the DEGs into three principal categories, namely, biological processes, cellular components, and molecular functions (Figure 6 and Supplementary Figure S5). For DEGs, which were considered responsible for drought tolerance (T vs. S), they showed highest numbers in three biological processes of protein phosphorylation, oxidation-reduction, and regulation of transcription (Figure 6). Most of the DEGs were associated with two cellular components, the membrane and nucleus. The protein binding, ATP binding, protein kinase activity, and DNA binding were the top four molecular functions for those DEGs (Figure 6). The same top categories in all three groups of the biological process, cellular component, and molecular function were found for the DEGs responsive to drought stress (T vs. T and S vs. S) (Supplementary Figure S5).

The reactome pathway analysis illustrated the top 30 pathways with the highest number of DEGs inside (Supplementary Figures S6, S7). The pathways of metabolism and regulation and amino acid metabolism were the most important pathways in both drought tolerance (T vs. S) and drought stress response (T vs. T and S vs. S) (Supplementary Figures S6, S7). In addition, for drought tolerance (T vs. S), the pathways of the cell cycle, cellular processes, and mitosis also showed a high number of genes (Supplementary Figure S6).

### Validation of Candidate Genes Using Quantitative Real-Time-PCR

**FIGURE 7:**
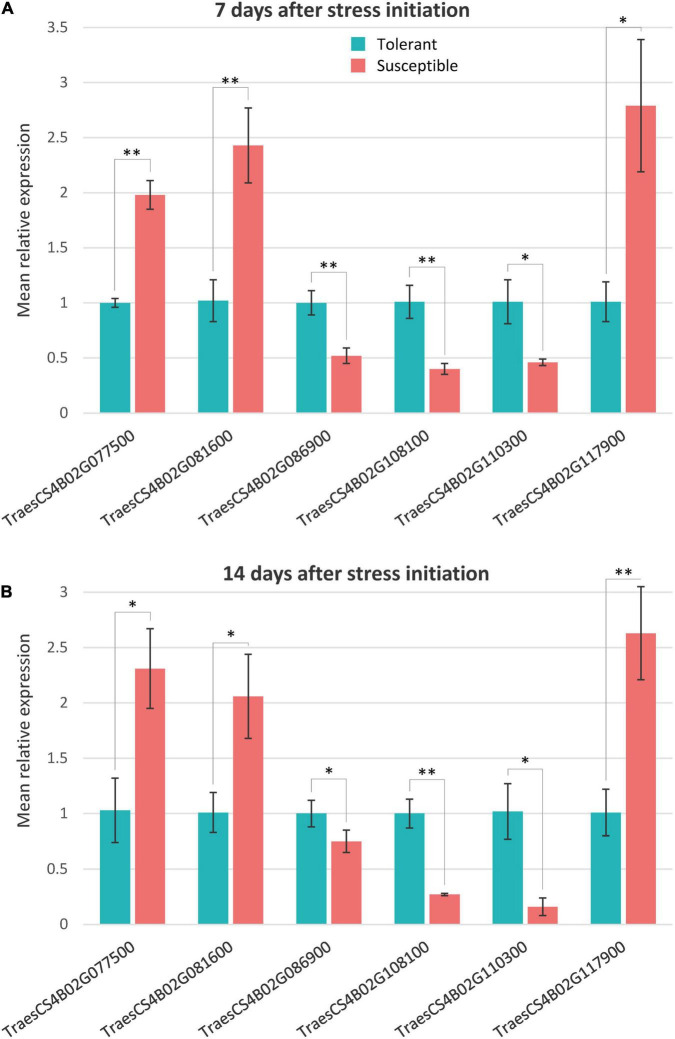
Quantitative real time PCR relative expression values of the selected genes in tolerant and susceptible isolines at panels **(A)** 7 days and **(B)** 14 days after stress initiation. Mean relative expression is the mean of NIL1 and NIL2. The expression data measured by subtracting the Ct number of the reference gene (*Actin*) from that of the target gene followed by calculation of 2^– ΔΔCT^. Values are means ± standard deviation, and the statistical significance was determined by two-sided *t*-test (*p < 0.05 and **p < 0.01).

The qRT-PCR expression analysis for the six candidate genes putatively responsible for drought tolerance in NIL1 and NIL2 under different treatments was conducted. The physical appearance of the amplification plot and the single distinct peak on the melt curve of each sample showed the reaction specificity of primers and high precision and efficiency of the PCR reaction (Supplementary Figure S8). The qRT-PCR expression of the six putative candidate genes showed significant differences between T and S isolines at both 7 and 14 days after stress initiation, and the expression patterns were consistent with those obtained from RNA-seq analysis (Figures 7A,B and Supplementary Table S6). The highly significant correlation (*r* = 0.96) between the qRT-PCR and RNA-seq data of expression ratios for these six genes demonstrated the reliability of the RNA-seq data in our study (Supplementary Figure S9).

## Discussion

Withholding water for 7 and 14 days after anthesis resulted in a significant reduction in the leaf RWC by 40% after 7 days and 70% after 14 days, signifying an effective and realistic degree of drought stress that resulted in a 35 and 70% reduction in yield in the tolerant isolines (when NIL1 and NIL2 were combined) and 44 and 78% reduction in yield in the susceptible isolines relative to the WW controls after 7 and 14 days without water, respectively. The T in comparison to the S isolines in both NIL1 and NIL2 maintained significantly higher chlorophyll content, chlorophyll fluorescence, RWC, dry weight of aerial parts, 1,000-kernel weight, and grain yield under stress conditions (Table 1).

Genes responsible for drought tolerance in the *qDSI.4B.1* QTL were detected by determining DEGs between T and S isolines under stress conditions, with special focus on the common DEGs between both NIL1 and NIL2. Accordingly, six common DEGs of *TraesCS4B02G110300, TraesCS4B02G086900, TraesCS4B02G108100 TraesCS4B02G077500, TraesCS4B02G081600*, and *TraesCS4B02G117900*, which were all located on 4BS, were found to be the main candidate genes for drought tolerance in *qDSI.4B.1* QTL (Table 3 and Supplementary Table S4). The two genes *TraesCS4B02G086900* and *TraesCS4B02G110300* of these six DEGs also had the SNP variants inside, which made them even more interesting candidates, showing not only different expression patterns but also the physical differences in their DNA sequences.

*TraesCS4B02G086900* with one SNP in the intron had higher expression in T isoline in both NIL pairs and both time points under stress conditions. The protein function of this gene is mainly described as the Transducin/WD40 repeat-like superfamily protein, which modulates various cellular processes, including plant stress and hormone responses (Xu et al., 2019). The various copies of the WD40 domain in this protein family fold into β-propeller arrangement that act as versatile scaffolds for protein-protein interactions (Mishra et al., 2012). In *Arabidopsis*, mutation of *XIW1* responsible for encoding a WD40 protein (XIW1) decreased drought resistance by reducing the induction of ABA-responsive genes (Xu et al., 2019). In another study, it was shown that a WD40 protein (HOS15) played a significant role in abiotic stress tolerance in plants by chromatin remodeling (caused by deacetylation of histone H4) (Zhu et al., 2008). The interaction between nonfermenting-1-related kinase and the WD40 repeat region of myoinositol polyphosphate 5-phosphatase (At1g05630) has been reported to have an essential role in developmental signaling, sugar metabolism, and stress tolerance in *Arabidopsis* (Ananieva et al., 2008). In mango, MiTTG1 (another WD40 protein) led to the formation of a ternary regulatory complex (MYB-bHLH-WD40) that resulted in higher adaptation to abiotic stresses by stimulating the growth of root hairs and increasing root length (Tan et al., 2021).

The small gene (1413 bp) *TraesCS4B02G110300* is a DEG with one SNP in exon, which showed higher expression in the T isoline in both NIL pairs and 7 and 14 days after stress imposition. The probable function of the *TraesCS4B02G110300* gene is acting as the elongation factor Ts (EF-Ts). The protein synthesis elongation factor Tu (EF-Tu) and EF-Ts are interacting proteins involved in the elongation stage of protein synthesis in plant organelles of mitochondria and plastids (Riis et al., 1990). In this process, EF-Ts facilitate the exchange of bound GDP for GTP in EF-Tu. This shapes the ternary complex of EF-Tu⋅GTP⋅aa-tRNA by binding EF-Tu⋅GTP with aminoacyl-tRNA, which leads to the location of this aminoacyl-tRNA at the A site of the ribosome for polypeptide elongation (Fu et al., 2012). Additionally, chaperone activity of EF-Tu gives it the binding ability to the hydrophobic regions of the denatured proteins to protect other proteins from aggregation caused by stress (Rao et al., 2004). The plastid protein translation, protein folding, retrograde signaling of stress responsive genes, in addition to chaperone activity, give EF-Tuan an important role in abiotic stress tolerance, such as heat tolerance (Li et al., 2018). The role of elongation factors, including EF-G, EF-Tu, and EF-1α in heat tolerance, has been reported in other studies (Bukovnik et al., 2009). The over-expression of chloroplast EF-Tu during grain filling is reported in spring wheat under high temperature and drought stress (Prasad et al., 2011). The addition of GTP and EF-Ts to EF-Tu by changing the conformation of the complex increased the refolding of denatured proteins, which can be a possible explanation for the role of EF-Ts in stress tolerance (Fu et al., 2012). However, the direct role of EF-Ts in stress tolerance, such as drought stress in plants, has not been previously reported.

*TraesCS4B02G108100* was found as a common DEG in both NIL pairs with higher expression in T isoline at both 7 and 14 days after stress initiation. This gene encodes ATP-dependent protease La (LON) domain-containing protein. LON is a serine protease from the AAA^+^ family located in the chloroplasts and mitochondria that plays an important role in cellular homeostasis under stress conditions (Li et al., 2010). Plant exposure to stress conditions such as drought stress can lead to the production of reactive oxygen species (ROS), damaging proteins by misfolding them through chemical modifications (Sachdev et al., 2021). These functionally impaired misfolded proteins generate toxic protein aggregates that interfere with normal cellular function (Rigas et al., 2014). Therefore, protein quality control plays an important role in defense against oxidative stress by organizing the assembly of the protein complex and breaking down misfolded or orphaned proteins (Lu et al., 2018). Chaperones and proteases are two opposing components of protein quality control by removing unfolded proteins from the cell (Sun et al., 2021). Chaperones prevent aggregation by facilitating the folding and assembly of newly synthesized proteins. In contrast, ATP-dependent proteases break down damaged and misfolded proteins and consequently reduce the number of non-functional proteins that might be generated by oxidative stress (Rigas et al., 2014). It is reported in *Arabidopsis* that the *atlon4* mutant, with the lack of a Lon protease (AtLon4), is more sensitive to drought stress than wild-type plants as a result of increased water loss, decreased water use efficiency, lower levels of ABA, and impaired stomatal closure (Li et al., 2010).

*TraesCS4B02G077500* is another common DEG in both NIL pairs that showed higher expression in the S isoline under drought stress. According to the wheat functional annotation v1.0 and the BLAST of the sequence, this gene encodes myosin-II heavy chain-like protein (MyHCs). However, myosin-II is mainly reported in animal cells and plant myosin falls only into two classes of VIII and XI (Reddy and Day, 2001). Myosins are molecular motors that interact with actin filaments to transport various cellular components by using chemical energy stored in ATP (Haraguchi et al., 2018). In plants, myosin (VIII and XI) has been reported to be involved in cell expansion and growth such as elongation and development of root hair cells, branches, trichome stalks, pavement cells, and stigmatic papillae (Ojangu et al., 2007, 2012; Duan and Tominaga, 2018). A study of *Arabidopsis* for the two class XI myosin mutants showed that, in the absence of this gene, salicylic acid stress resulted in reduced root length, although it did not have any effect on root length under heat stress (George, 2011). Another study on *Arabidopsis* provided genetic evidence for the role of XI myosin in flower morphogenesis and leaf longevity through its contribution to auxin responses, stress-induced senescence, and cell death (Ojangu et al., 2018). Bao et al. (2020) showed that the plant-specific gene, *constitutively stressed 1* (*COST1*), that produces the COST1 protein (also annotated as a myosin-IV-like protein) negatively regulated drought resistance by direct regulation of autophagy in *Arabidopsis*. The defect of the gene in the *cost1* mutant reduced the growth and enhanced the drought tolerance *via* constitutive autophagy and increased the expression of the drought-responsive genes. On the other hand, the overexpression of *COST1* results in drought hypersensitivity and decreased autophagy. The proposed working model is that, in optimal conditions, COST1 allows plant growth by repressing autophagy. However, the degradation of COST1 under drought stress leads to activation of autophagy and suppression of growth to enhance drought tolerance (Bao et al., 2020).

The other common DEG between both NIL pairs is *TraesCS4B02G081600* that showed higher expression in S isoline under drought stress. This gene encodes a protein containing the B3 DNA-binding domain (DBD). The B3 domain is a highly conserved domain exclusively found in transcription factors (TFs), consisting seven β-barrels and two short α-helices (100–120 residues) to form a DNA-binding pseudobarrel protein fold (Zhao et al., 2017). The B3 superfamily is classified into four gene families of ARF (auxin response factor), LAV [leafy cotyledon2 (LEC2)-abscisic acid insensitive3 (ABI3)-val], related to ABI3/VP1 (RAV), and REM (reproductive meristem) (Xia et al., 2019). The ARF has been shown to be implicated in senescence, hormone signaling, development, and abiotic stress responses through regulating the expression of auxin-responsive genes by binding to auxin-responsive elements (AuxREs; TGTCTC) located upstream of these genes (Kang et al., 2018). Abscisic acid (ABA) has implications in the regulation of seed dormancy, leaf senescence, stomatal conductance, and adaptation to various stresses (Sah et al., 2016). The regulatory genes of abscisic acid-insensitive3 (ABI3), FUSCA3 (FUS3), and leafy cotyledon1 (LEC1) play an important role in ABA signaling during seed maturation (Sun et al., 2017). The RAV transcription factors with B3 and APETALA2 (AP2) play a critical role in plant growth regulation and development, and responses to abiotic stress (Wang et al., 2020). It has been reported that RAV1, RAV1L, TEM1/EDF1, and RAV2/TEM2 have negative regulatory effects on organ senescence and abiotic stresses (Fu et al., 2014; Chen et al., 2015; Xia et al., 2019).

*TraesCS4B02G117900* that encodes a 54-kDa protein subunit of the signal recognition particle (SRP54) was found as another candidate gene for drought tolerance. This gene was common in both NIL pairs and had the higher expression in S isoline in all comparisons. Targeting of proteins to appropriate sub-cellular compartments is an essential process in all living organisms (Janda et al., 2010). The signal recognition particle (SRP) made of an RNA and at least one polypeptide of ∼54 kDa (SRP54) has a vital role in targeting secretory proteins in the rough endoplasmic reticulum (ER) of eukaryotic cells (Supplementary Figure S10; Schünemann, 2004). The SRP54 mediates the binding to the signal peptide and contains two domains: an amino-terminal domain that has a putative GTP-binding site (G-domain) and a carboxy-terminal domain that contains a high abundance of methionine residues (M-domain) (Zopf et al., 1990). In *Arabidopsis*, the mutant *chaos* impaired the chloroplast-recognition particle (cpSRP43) coding gene, and demonstrated significantly higher tolerance to photooxidative stress in both laboratory and field conditions. This tolerance was related to lower production of H_2_O_2_, lower ascorbate levels, and better photosynthetic performance that led to lower photooxidative damage together with faster growth recovery in young seedlings and higher survival rates (Klenell et al., 2005). Signal recognition particle receptor α was detected as an associated gene with drought-stress response and tolerance at different growth stages of the rice plant (Gorantla et al., 2007). However, the precise mechanism and the role of the *SRP54* gene in stress tolerance remain to be investigated.

The expression of the six putative candidate genes was further investigated in the public transcriptomic database using an expVIP virtual machine.^3^ Among the six genes, *TraesCS4B02G086900* and *TraesCS4B02G117900* showed the highest and *TraesCS4B02G081600* the lowest expression in spikes, roots, shoots, and leaves in the seedling, vegetative, and reproductive stages under abiotic stresses (Supplementary Figure S11). Under abiotic stresses, *TraesCS4B02G077500* exhibited higher expression in spikes at the reproductive stage in comparison to roots, leaves, and shoots at seedling and vegetative stages. *TraesCS4B02G108100* and *TraesCS4B02G110300* both had their highest expression in leaves and shoots and their lowest expression in spikes and roots under abiotic stresses. *TraesCS4B02G081600* under abiotic stresses had its highest expression in spikes at the reproductive stage, followed by leaves and shoots at the seedling stage (Supplementary Figure S11). These results further confirmed the importance of these six putative candidate genes under abiotic stress in wheat.

The wheat’s 4BS is an important genomic location for yield and yield-related traits (Liu et al., 2020; Zhang et al., 2021). The *reduced height-1* (*Rht1*) gene associated with the green revolution is located in ∼30 Mbp of 4BS (Figure 5). The *Rht1* gene reduces plant height by decreasing the ability of plants to respond to gibberellic acid (GA) (Jobson et al., 2019), resulting in increased yields from reduced lodging, particularly when irrigated and fertilized, and more assimilates being translocated to the grain rather than being utilized for height growth. A previous meta-QTL (MQTL) analysis for yield and yield components mapped three refined major locations of MQTL4B.1, MQTL4B.2, and MQTL4B.3, containing four, five, and five QTLs, respectively (Figure 5). These QTLs were responsible for grain number, spike number, and thousand-grain weight in all studied environmental conditions (Liu et al., 2020). According to the number of DEGs and genes containing SNPs/indels, we confined the *qDSI.4B.1* QTL to an interval of about 88 Mbp from 49 to 137 Mbp of 4BS as the most probable location for this locus (Figure 5). Identifying the novel candidate genes underlying *qDSI.4B.1* QTL indicates that, apart from *Rht-B1* gene that has been reported to have pleiotropic effect on grain yield under non-stress conditions (Balyan and Singh, 1994), there are other genes located on 4BS responsible for mechanisms affecting grain yield under drought stress.

The responsive genes to drought stress in T and S isolines were detected by the comparison of each isoline in the two different treatments, followed by finding common DEGs between all T_v_T and S_v_S comparisons (Figure 2 and Supplementary Table S3). In the drought-responsive genes in the T isolines, the number of upregulated DEGs was around 4 times (623 to 142) the number of downregulated genes (Figure 2). Under drought stress, we witnessed the upregulation in tolerant isolines of the genes of the low/high molecular weight glutenin subunit, α- and γ-gliadin. Gliadins and glutenins are the two main components of the gluten that determine the bread quality (Phakela et al., 2021). The upregulation of the transcription factors (TFs) of AP2-like ethylene-responsive (AP2/ERF), bZIP, MYB, and NAC domain-containing protein was also observed under drought stress in tolerant isolines. TFs are important components of the gene regulatory networks involved in plant responses to stress, and their expression, function, and regulation have been extensively studied in wheat and other plants (Zhao et al., 2018; Castelán-Muñoz et al., 2019; Meraj et al., 2020). Cytochrome P450s (CYPs) protect plants from abiotic and biotic stresses through biosynthesis of secondary metabolites, antioxidants (e.g., carotenoids and flavonoids) and phytohormones (e.g., abscisic acid) (Pandian et al., 2020). The upregulation of CYPs under osmotic stress has been reported in many plants, including rice, sorghum, and *Arabidopsis* (Gorantla et al., 2007; Johnson et al., 2014; Rao et al., 2020). Plant heat shock proteins (HSPs) have a key role in conferring biotic and abiotic stress tolerance (Tian et al., 2021). HSPs enhance membrane stability by regulating the antioxidant enzyme system. Moreover, HSPs as chaperones, play roles in protein folding, transportation, localization, and degradation of non-native proteins (Ul Haq et al., 2019). The up or downregulation of HSPs in drought stress has been reported in several studies (Reddy et al., 2014; Singh et al., 2016; Xiang et al., 2018). NBS-LRR-like (nucleotide binding, leucine-rich repeat) resistance protein belongs to the largest disease resistance gene family in plants with a central role in biotic and, in some cases, abiotic stresses such as drought (Van Ghelder et al., 2019). Interestingly, we witnessed downregulation of all NBS-LRRs disease resistance genes (both TIR and non-TIR subfamilies) in T isolines (Figure 3 and Supplementary Table S3). In a transcriptomic study of *Picea glauca*, the majority of differentially expressed NBS-LRRs were downregulated after several days of water deprivation (Van Ghelder et al., 2019). The reduction in the expression of NBS-LRR-like resistance proteins can be one of the reasons for higher susceptibility to pathogens in drought stress condition (Sinha et al., 2019).

## Conclusion

For a better understanding of the wheat response to drought stress and, especially, deciphering the role of *qDSI.4B.1* QTL as a major genomic region harboring effective genes for drought tolerance, functional studies through RNA-seq analysis were conducted under moderate and severe water stress on two NIL pairs that were produced from crossing between C306 and Dharwar Dry varieties. The comparison of the gene expression pattern and variant calling between T and S isolines led to the detection of novel candidate genes, conferring drought tolerance in *qDSI.4B.1* location. This indicates that, apart from important *Rht-B1* genes that have an effect on grain yield under non-stress condition, there are other genes on the 4BS chromosome affecting grain yield under drought stress. Accordingly, the target QTL was confirmed as a critical genomic region for drought tolerance-containing genes involved in gene regulation, cell elongation, protein quality control, secondary metabolism, and auxin and ABA signaling. qRT-PCR analysis confirmed the six identified major genes by RNA-seq. However, complementary studies utilizing transgenic approaches may be required to clarify the function of the candidate genes. According to our findings, we suggest the most probable location for the *qDSI.4B.1* QTL is between 49 and 137 Mbp of the 4BS chromosome. The SNP and indel markers within the QTL interval showed consistent distinguishable alleles between contrasting isolines that can be further used for delineation of the locus to smaller intervals through fine mapping. Overall, we consider that this study’s outcome can be valuable for understanding the mechanism of drought tolerance in wheat and, ultimately, for the breeding of drought-tolerant genotypes.

## Data Availability Statement

The datasets presented in this study can be found in online repositories. The names of the repository/repositories and accession number(s) can be found in the article/Supplementary Material.

## Author Contributions

SN: conceptualization, methodology, data curation, formal analysis, and writing – original draft. MM: conceptualization, methodology, resources, supervision, and writing – review and editing. HL: and GY conceptualization, funding acquisition, methodology, resources, supervision, and writing – review and editing. NT: conceptualization, methodology, supervision, and writing – review and editing. All authors contributed to the article and approved the submitted version.

## Conflict of Interest

The authors declare that the research was conducted in the absence of any commercial or financial relationships that could be construed as a potential conflict of interest.

## Publisher’s Note

All claims expressed in this article are solely those of the authors and do not necessarily represent those of their affiliated organizations, or those of the publisher, the editors and the reviewers. Any product that may be evaluated in this article, or claim that may be made by its manufacturer, is not guaranteed or endorsed by the publisher.

## Footnotes

1
https://www.ncbi.nlm.nih.gov/bioproject/PRJNA760243

2
https://wheat-urgi.versailles.inra.fr/

3
http://www.wheat-expression.com

## Glossary

2OG, 2-oxoglutarate; AP2/ERF, AP2-like ethylene-responsive; 4BS, short arms of chromosome 4B; 7 d, 7 days after stress; 14 d, 14 days after stress; AAA+, ATPases associated with diverse cellular activities; ABA, Abscisic acid; ABI3, abscisic acid-insensitive 3; AGRF, Australian genome research facility; AP2, APETALA2; ARF, auxin response factor; ATP, adenosine triphosphate; AuxREs, auxin-responsive elements; bHLH, basic helix–loop–helix; bZIP, basic leucine zipper; C, control; cDNA, complementary DNA; CMP, count per million; COST1, constitutively stressed 1; cpSRP43, chloroplast signal recognition particle 43; CYPs, Cytochrome P450s; DBD, B3 DNA binding domain; DEG; differentially expressed gene; DS, drought stress; DSI, drought susceptibility index; DW, dry weight; EDF, ethylene response DNA-binding factor; EF-1α, translation elongation factor 1A; EF-Ts, elongation factor Ts; EF-Tu, elongation factor Tu; ER, endoplasmic reticulum; FAO, food and agriculture organization; FDR, false discovery rate; Fm, maximum fluorescence; FPKM, fragments per kilobase of exon per million mapped reads; FUS3, FUSCA3; Fv, variable fluorescence; FW, fresh weight; G × E, genetic environment interaction; GA, gibberellic acid; GB, gigabyte; G-domain, GTP-binding site; GDP, guanosine diphosphate; GO, gene ontology; GRS, general stress response genes; GTP, guanosine triphosphate; GY, grain yield; H_2_O_2_, hydrogen peroxide; HI, harvest index; HIF, heterogeneous inbreed family; HOS15, high expression of osmotically responsive genes 15; HSPs, plant heat shock proteins; jndel, insertion and deletion; IWGSC, international wheat genome sequence consortium; kDa, kilodalton; LAV, leafy cotyledon2-abscisic acid insensitive3-val; LEC1, leafy cotyledon1; LEC2, leafy cotyledon2; LON, ATP-dependent protease La; Mbp, mega base pairs; M-domain, methionine residues; MDS, multidimensional scaling plot; MiTTG1, mango transparent testa glabra 1; MQTL, meta-QTL; MYB, myeloblastosis; MyHCs, myosin-2 heavy chain-like protein; NBS-LRR, nucleotide-binding site leucine-rich repeat; NCBI, national center for biotechnology information; NIL, near isogenic line; NS, not significant; PEA, pocket plant efficiency analyzer; PH, plant height; PC, pot capacity; Q30, quality score of 30; qRT-PCR, quantitative real time polymerase chain reaction; QTL, quantitative trait locus; RABT, reference annotation-based assembly; RAV, related to ABI3/VP1; RB, root biomass; REM, reproductive meristem; Rht1, reduced height-1; RILs, recombinant inbred lines; RIN, RNA integrity number; RNA-seq, RNA sequencing; ROS, reactive oxygen species; RWC, relative water content; S, susceptible; SB, shoot biomass, SNP, single nucleotide polymorphisms; SPAD, the soil plant analysis development; SRA, sequence read archive; SRP, signal recognition particle; T, tolerant; TEM, TEMPRANILLO; TFs, transcription factors; TIR, toll/interleukin-1 receptor-like; TMM, weighted trimmed mean of M-values; TPD1, tapetum determinant 1; tRNA, transfer ribonucleic acid; UTR, untranslated region; V, versus; WD40, transducin/WD40 repeat-like superfamily protein; WW, well water; XIW1, XPO1-interacting WD40 protein 1; ΔΔCT, delta delta C (T) method.
