# Supplementary material for: Transcriptome Analyses of Near Isogenic Lines Reveal Putative Drought Tolerance Controlling Genes in Wheat
Source: Front Plant Sci. 2022 Mar 29;13:857829. doi: 10.3389/fpls.2022.857829 (PMC9005202; doi:10.3389/fpls.2022.857829)
Supplement: Supplementary file 1 [file Data_Sheet_1.pdf]

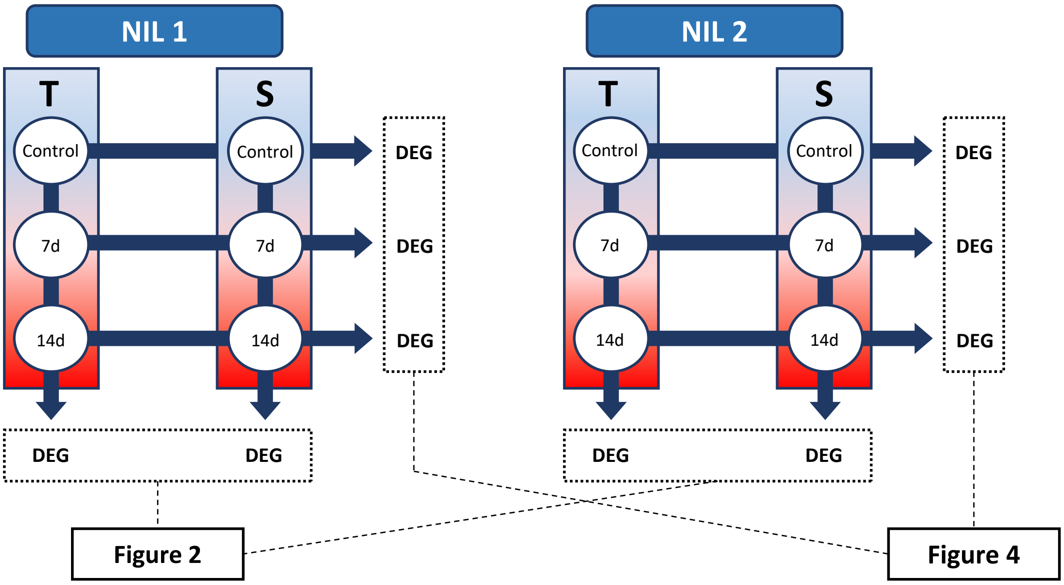

Supplementary Figure 1: Experiment design for differentially expressed genes (DEGs) analysis. ‘7d’ and ’14d’ represent 7 days and 14 days after stress induction, respectively. ‘T’ means tolerant and ‘S’ means susceptible isoline.

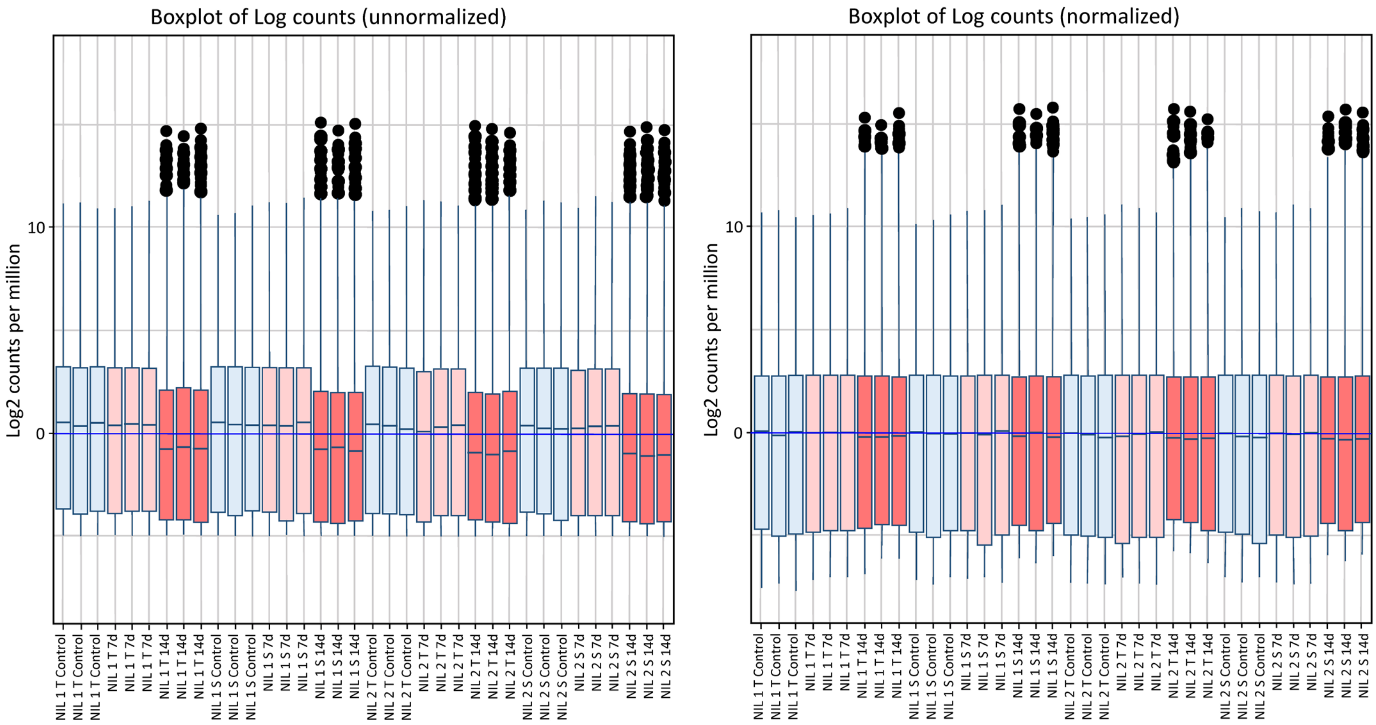

Supplementary Figure 2: Normalized (right) and unnormalized (left) expression boxplots showing the distribution of expression values in 36 samples. ‘7d’ and ’14d’ represent 7 days and 14 days after stress induction, respectively. ‘T’ means tolerant and ‘S’ means susceptible isoline. Different colours show different treatments.

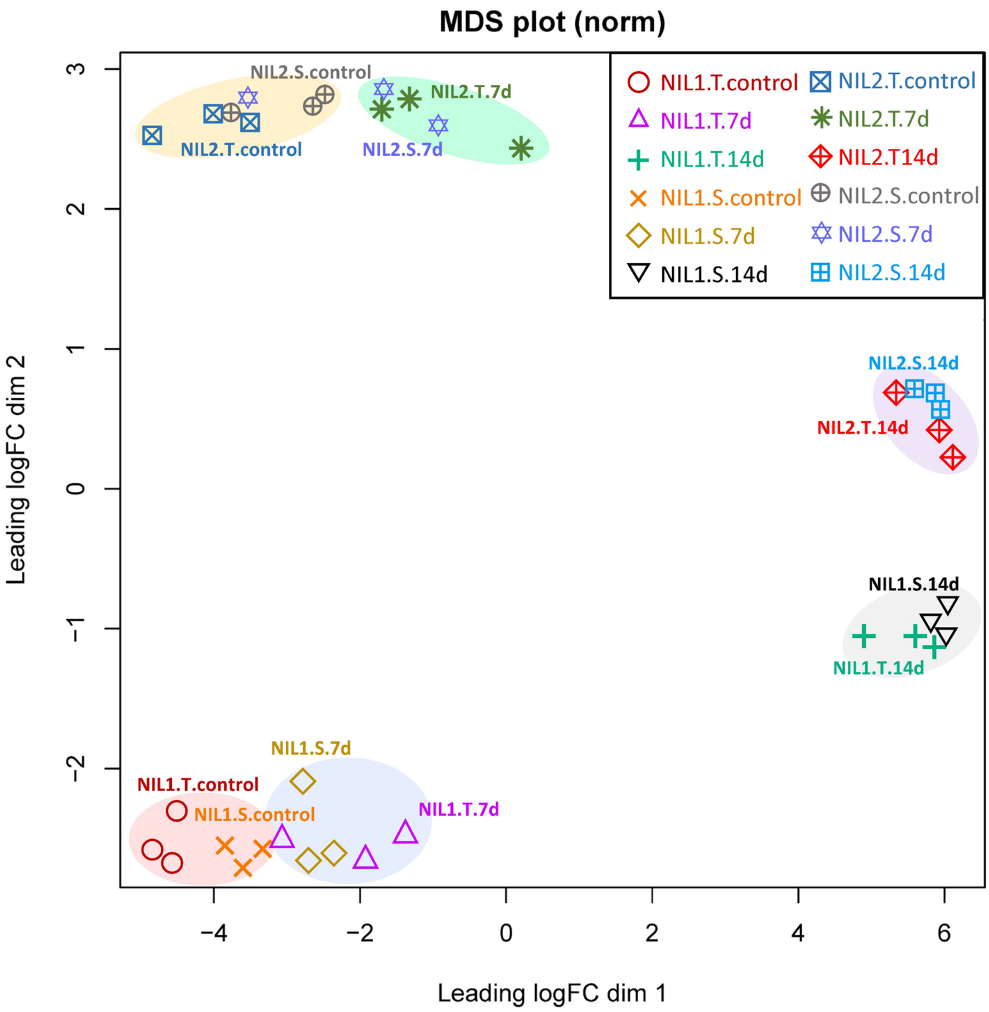

Supplementary Figure 3: Multidimensional scaling plot (MDS) showing distances between the samples correspond to the leading log fold change of RNA-seq samples. The leading logFC (base 2 logarithm of fold change) is the average of the largest absolute logFC between each pair of samples. ‘7d’ and ’14d’ represent 7 days and 14 days after stress induction, respectively. ‘T’ means tolerant and ‘S’ means susceptible isoline.

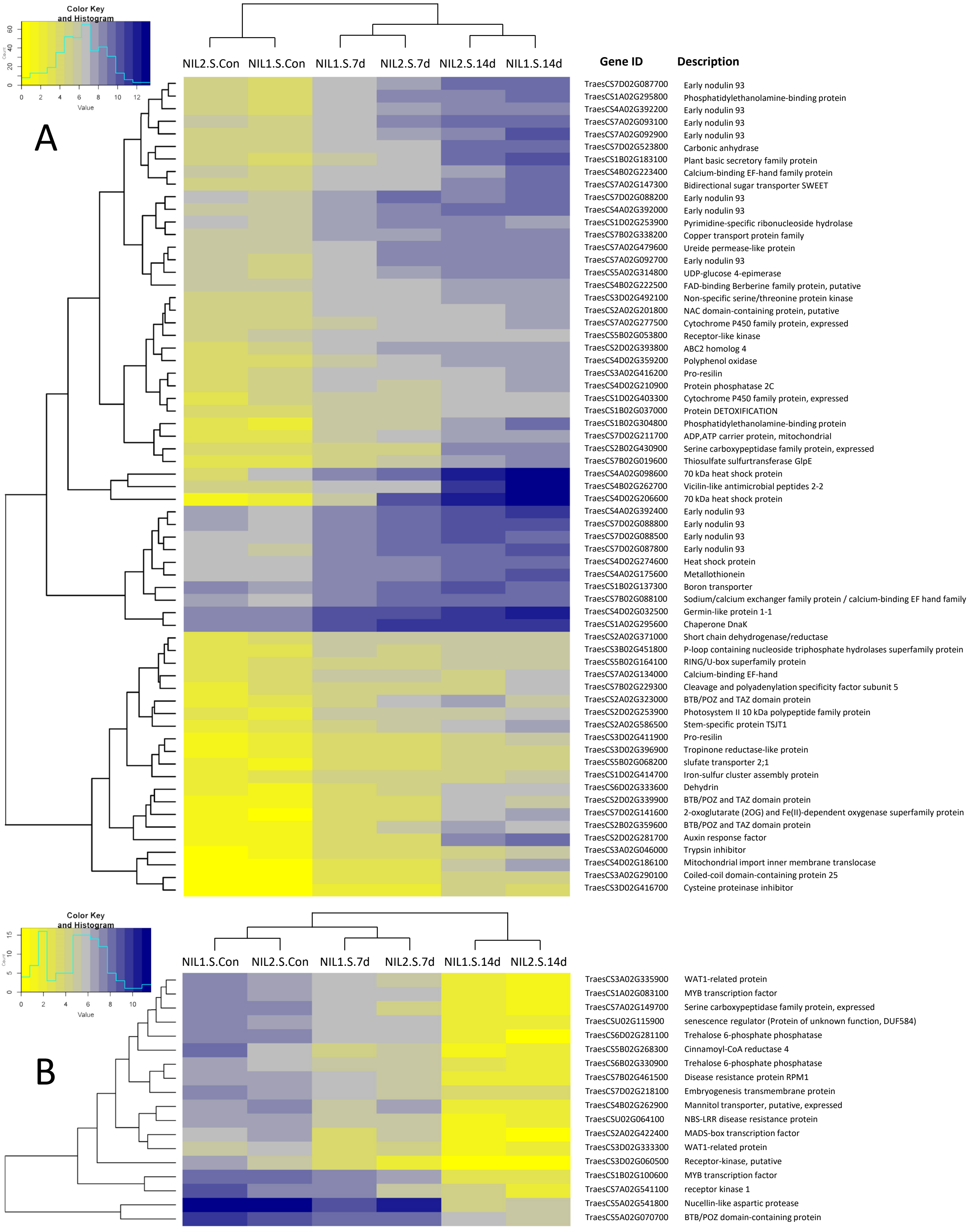

Supplementary Figure 4: Heatmaps showing expression and clustering of A) up-regulated and B) down-regulated genes responsive to drought stress in the susceptible isolines. Colour keys represent the log2 of normalized expression values and histogram of the counts. Each row represents a gene and each column a sample. Symbols are 'Con' for control; 'S' for susceptible isoline; '7d' and '14d' for 7days and 14days after stress induction, respectively. The DEGs were determined with the threshold of FDR ≤ 0.05 and the absolute value of log2 fold change ≥ 1 or ≤ -1.

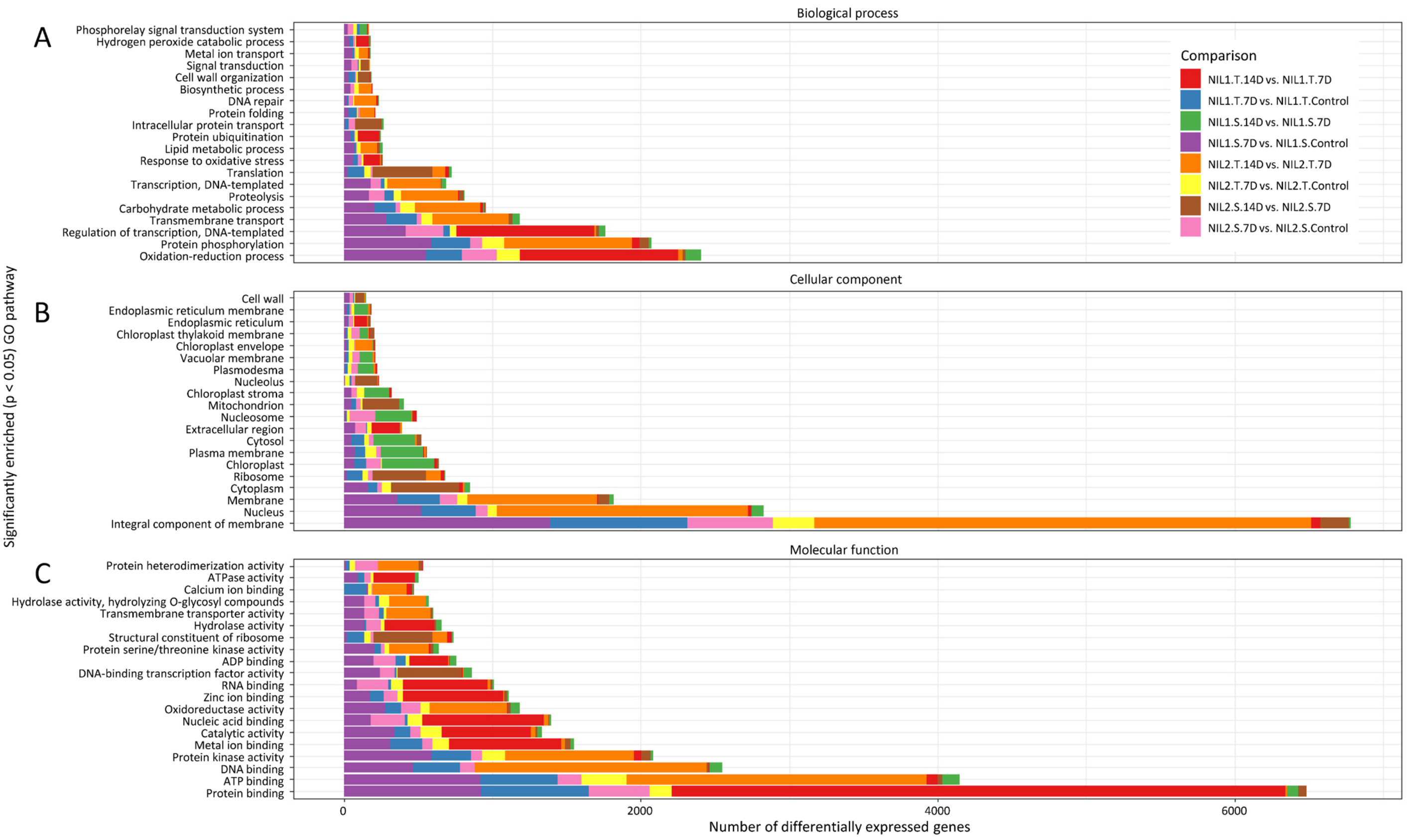

Supplementary Figure 5: Gene ontology analysis (GO) of the differentially expressed genes (DEGs) identified when comparing different treatments for the same isoline (T vs T and S vs S). Top significantly enriched pathways in A) biological process, B) cellular component, and C) molecular function, are illustrated with *p*-value < 0.05. Symbols are 'T' for tolerant isoline; 'S' for susceptible isoline; '7D' and '14D' for 7 days and 14 days after stress induction, respectively.

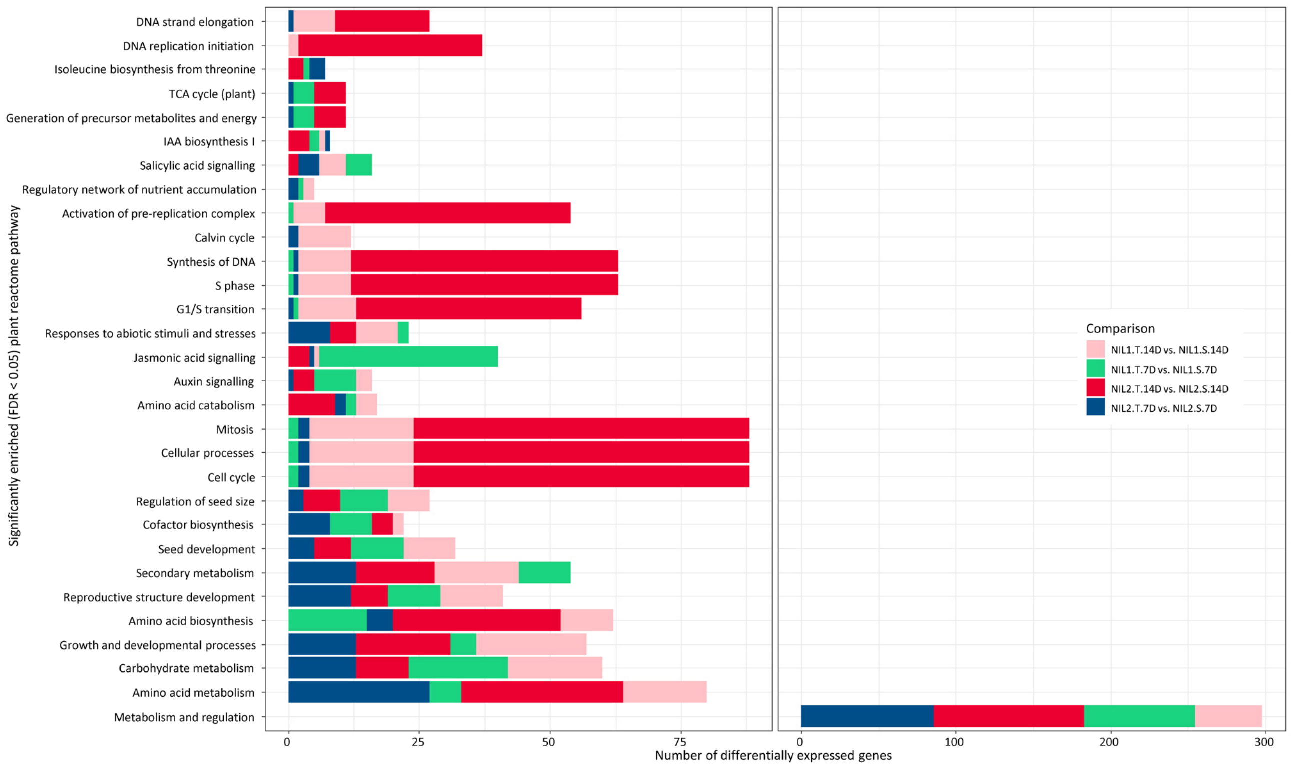

Supplementary Figure 6: Reactome pathway analysis of the differentially expressed genes (DEGs) identified when comparing tolerant and susceptible isolines (T vs S). Top significantly enriched pathways are illustrated with FDR < 0.05. Symbols are 'T' for tolerant isoline; 'S' for susceptible isoline; '7D' and '14D' for 7 days and 14 days after stress induction, respectively.

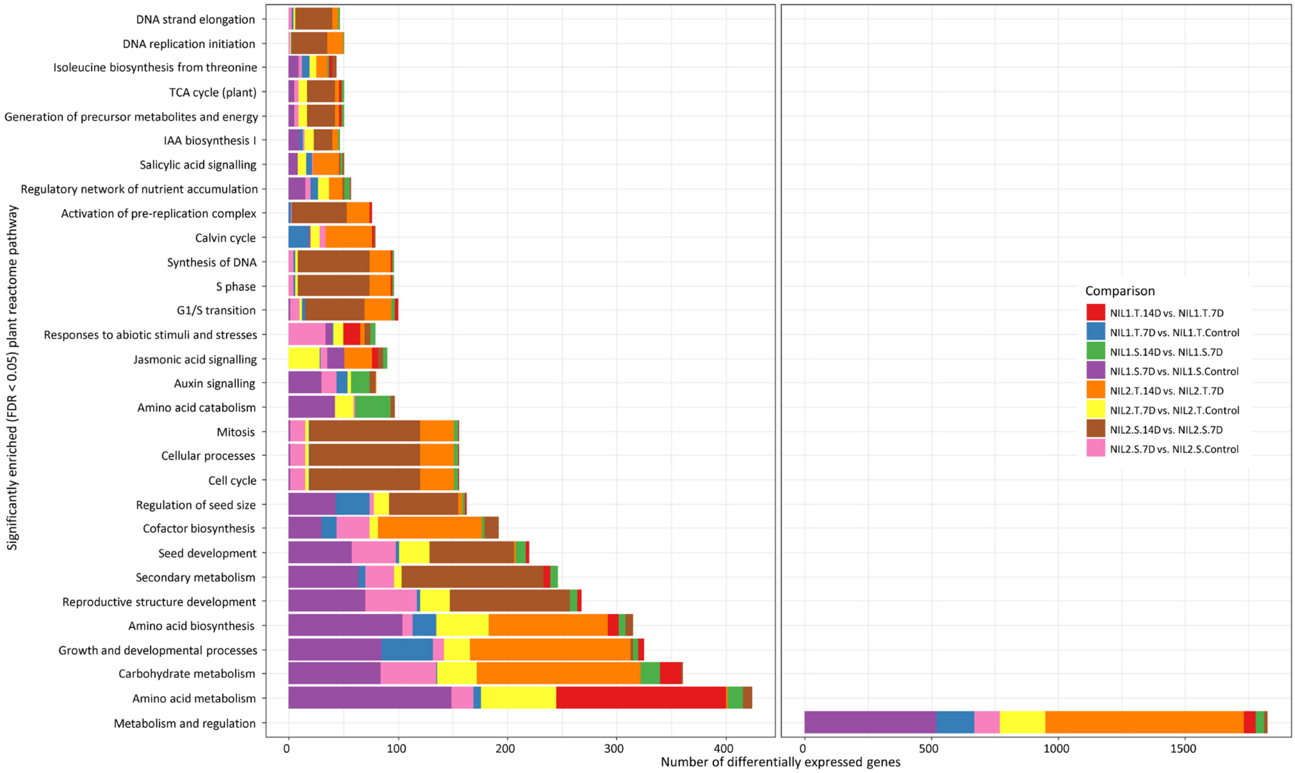

Supplementary Figure 7: Reactome pathway analysis of the differentially expressed genes (DEGs) identified when comparing different treatments for the same isoline (T vs T and S vs S). Top significantly enriched pathways are illustrated with FDR < 0.05. Symbols are 'T' for tolerant isoline; 'S' for susceptible isoline; '7D' and '14D' for 7 days and 14 days after stress induction, respectively.

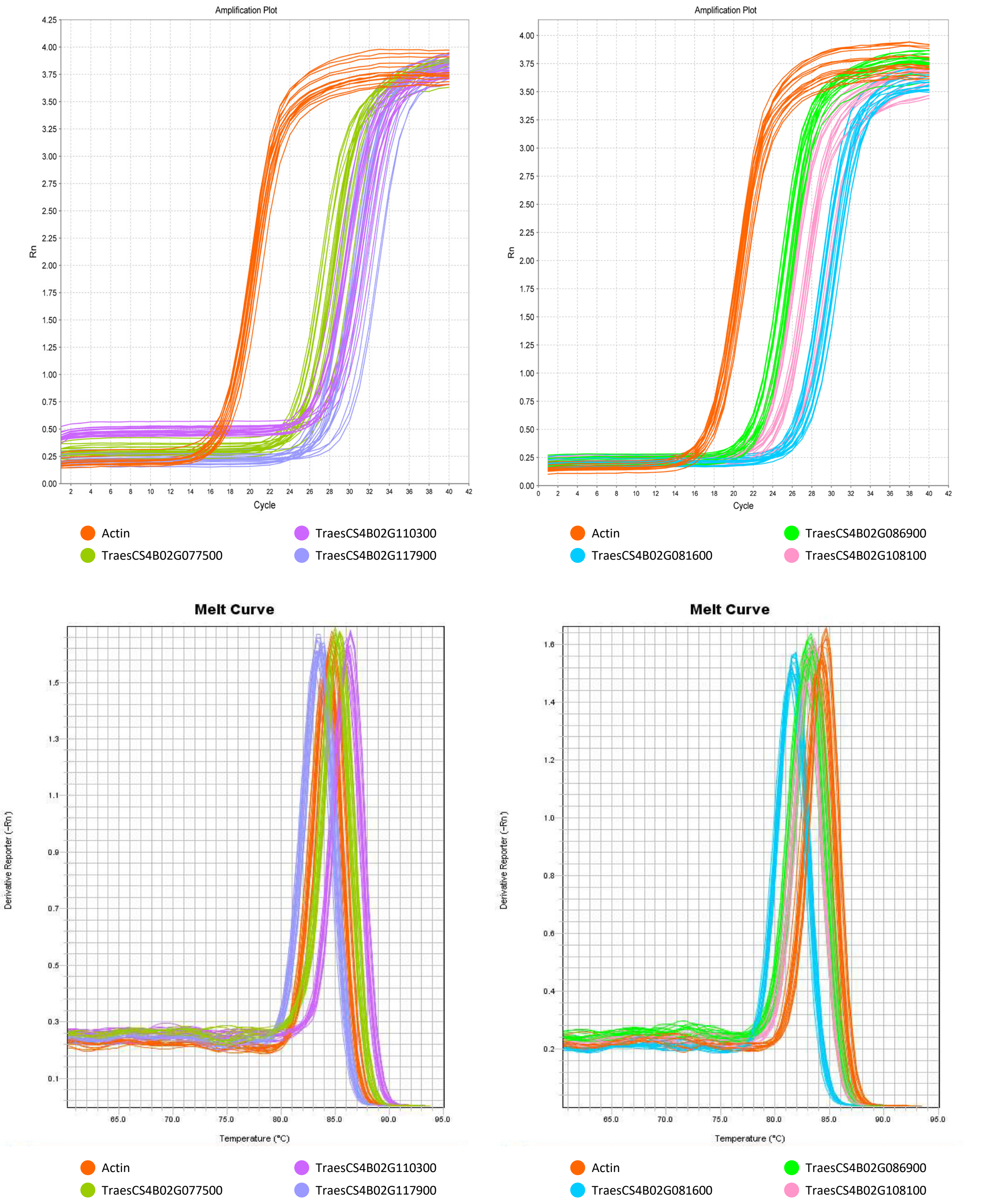

Supplementary Figure 8: Quantitative real-time PCR amplification plot and melt curve of the six selected genes on wheat 4BS using the comparative CT method ($2^{-\Delta\Delta CT}$). *Actin* was used as an internal housekeeping reference genes. Single distinct peak for melt curve of each sample shows formation of no primer-dimer artifact and ensures the reaction specificity (only targeted amplicon).

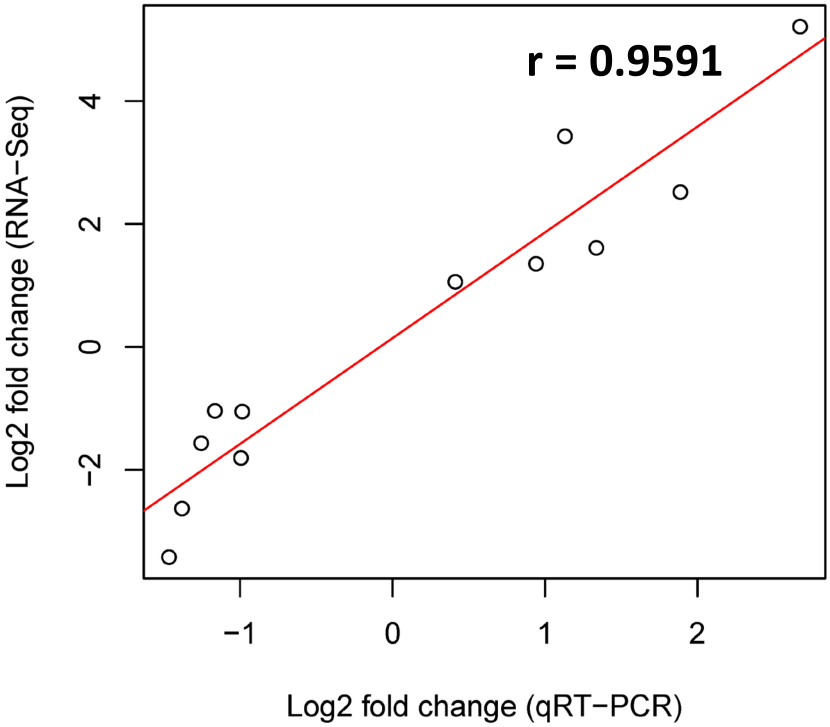

Supplementary Figure 9: Correlation between the RNA sequencing (RNA-Seq) and quantitative real-time PCR (qRT-PCR) data of the expression ratios for the six selected genes (i.e. each point in the chart is T7d/S7d or T14d/S14d).

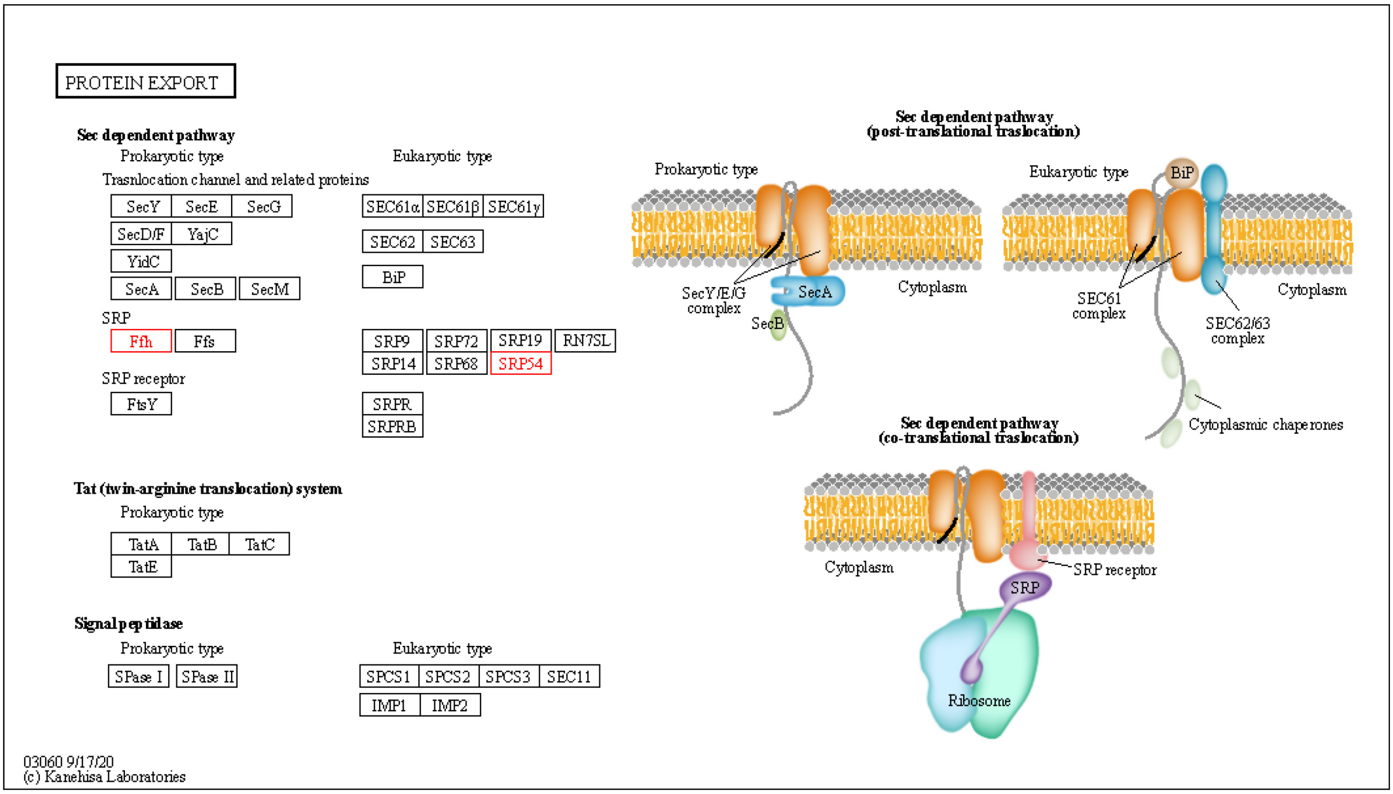

Supplementary Figure 10: The protein export pathway (secretion system) derived from Kyoto encyclopedia of genes and genomes (KEGG) (http://www.kegg.jp/). The sec dependent pathway is the general protein export system that transports newly synthesized proteins into or across the cell membrane. The red box shows the signal recognition particle subunit (SRP54) which is product of *TraesCS4B02G117900* as one of the six candidate genes responsible for drought tolerance.

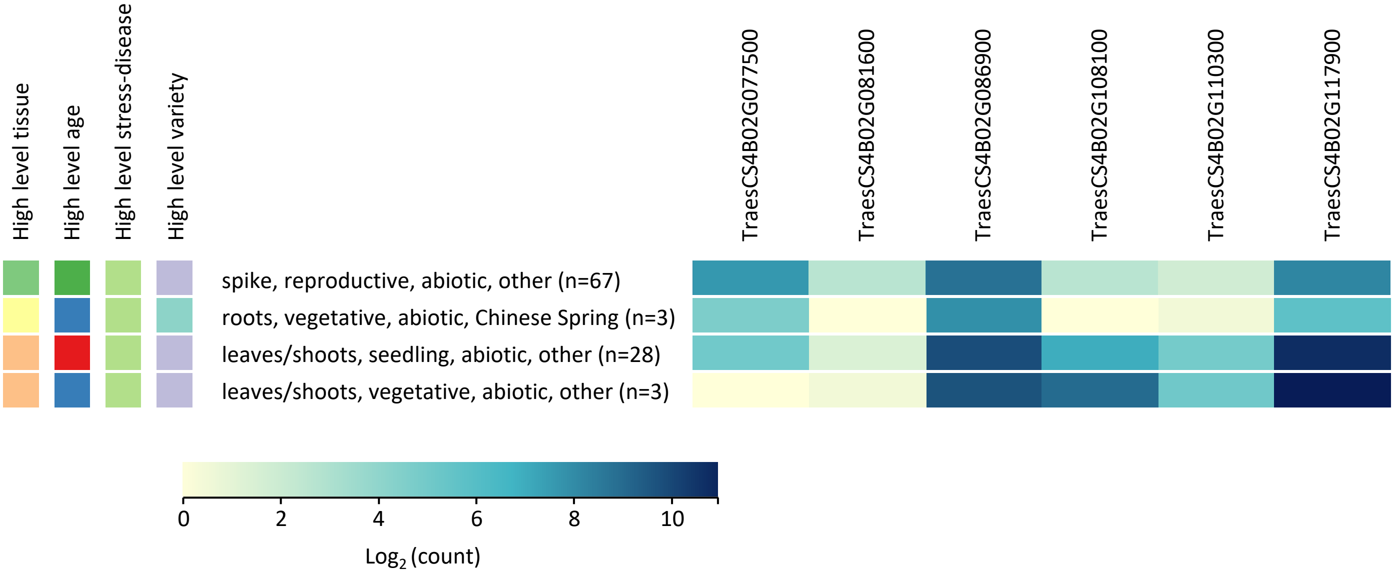

Supplementary Figure 11: Heatmap showing the expression of the six putative candidate genes under abiotic stresses through analysis in expVIP virtual machine (http://www.wheat-expression.com) using the public transcriptomic database of wheat. Colours represent the log2 of the count and "n" is RNA sequencing replicates used to generate the heatmap expression values.
